# Supplementary material for: Human Oxygenase Variants Employing a Single Protein FeII Ligand Are Catalytically Active
Source: Angew Chem Weinheim Bergstr Ger. 2021 May 19;133(26):14778–84. doi: 10.1002/ange.202103711 (PMC10947486; doi:10.1002/ange.202103711)
Supplement: Supplementary file 1 — Supplementary [file ANGE-133-14778-s001.pdf]

Angewandte Chemie
Eine Zeitschrift der Gesellschaft Deutscher Chemiker

Supporting Information

Human Oxygenase Variants Employing a Single Protein Fe^{II} Ligand Are Catalytically Active

*Amelia Brasnett[+], Inga Pfeffer[+], Lennart Brewitz[+], Rasheduzzaman Chowdhury, Yu Nakashima, Anthony Tumber, Michael A. McDonough, and Christopher J. Schofield**

ange_202103711_sm_miscellaneous_information.pdf

Supporting Information

Table of contents

1. Supporting figures and tables S2-S29
2. AspH variant inhibition assays S30
3. Crystallography S31
4. References S31-S32

1. Supporting figures and tables

Supporting Figure S1. The H679A and H725A variants of human *N*-terminally His$_6$-tagged AspH$_{315-758}$ were obtained in highly purified form as analysed by sodium dodecyl sulfate polyacrylamide gel electrophoresis (SDS-PAGE) with Coomassie staining and MS analysis (continues on the following page).

Site-directed mutagenesis to obtain plasmid DNA coding for the H679A and H725A variants of the reported human wildtype (wt) *N*-terminal His$_6$-AspH$_{315-758}$ (His$_6$-AspH$_{315-758}$) construct[1] were carried out using the QuikChange (Stratagene) site-directed mutagenesis kit according to the manufacturer's instructions. For PCR experiments, high fidelity PfuTurbo DNA polymerase (Stratagene) was used, primers were designed for the H679A AspH variant (forward primer: 5′-CTCACGTGTGGCCGGCCACAGGGCCC-3′, reversed primer: 5′-GGGCCCTGTGGCCGGCCACACGTGAG-3′) and for the H725A AspH variant (forward primer: 5′-GATGACTCCTTTGAGGCCGAGGTATGGCAGG-3′, reversed primer: 5′-CCTGCCATACCTCGGCCTCAAAGGAGTCATC-3′).

AspH variants were produced according to our reported procedure for wt His$_6$-AspH$_{315-758}$.[1] A pET-28a(+) vector encoding for the H679A or H725A His$_6$-AspH$_{315-758}$ variants was transformed into *E. coli* BL21 (DE3) cells. The resultant cells were grown in 2TY media supplemented with kanamycin (0.05 mM) at 37 °C with shaking (180 rpm). AspH production was induced at an OD$_{600}$ of ~1.2 at 18 °C by adding isopropyl ß-D-thiogalactopyranoside (1 M) to a final concentration of 0.1 mM. Cells were shaken for 16 h at 18 °C (180 rpm) and harvested by centrifugation (8000 rpm, 8 min, 4 °C); the resultant cell pellets were stored at -80 °C. Frozen cells were resuspended (30 g / 100 mL) in ice-cold 50 mM HEPES buffer (pH 7.5, 500 mM NaCl, 5 mM imidazole) containing EDTA-free protease inhibitor cocktail tablets (1 tablet/50 mL; Roche Diagnostics or Sigma-Aldrich) and DNAse I (bovine pancreas, grade II, Roche Diagnostics). The cells were lysed by sonication on ice (8x30 sec bursts; Sonics Vibra-Cell VCX500, amplitude: 60%), the lysates were then centrifuged (20000 rpm, 30 min, 4 °C). The supernatant containing AspH was purified at 4 °C by Ni(II)-affinity chromatography (HisTrap HP column, GE Healthcare; 1 mL/min flow rate) using an ÄKTA Pure machine (GE Healthcare) with a gradient of wash (50 mM HEPES, pH 7.5, 500 mM NaCl, 40 mM imidazole) and elution buffers (50 mM HEPES, pH 7.5, 500 mM NaCl, 500 mM imidazole). Eluted fractions containing AspH were pooled, then concentrated using an Amicon Ultra centrifugal filter (4000 rpm, 4 °C), and further purified by size-exclusion chromatography using a HiLoad 26/60 Superdex 75 pg 300 mL column with a flow rate of 1 mL/min and 50 mM HEPES (pH 7.5, 150 mM NaCl) as elution buffer. AspH variants were >95% pure by SDS-PAGE analysis and had the anticipated mass. The H679A and H725A variants of His$_6$-AspH$_{315-758}$ were stored in 50 mM HEPES buffer (pH 7.5, 150 mM NaCl) at a concentration of approximately 100 μM at -78 °C; fresh aliquots were used for all biochemical experiments.

(**a**) 10-12% SDS-PAGE analysis of the H679A and H725A His$_6$-AspH$_{315-758}$ variants after Ni(II)-affinity chromatography (HisTrap column) and subsequent size-exclusion chromatography (Superdex 75).

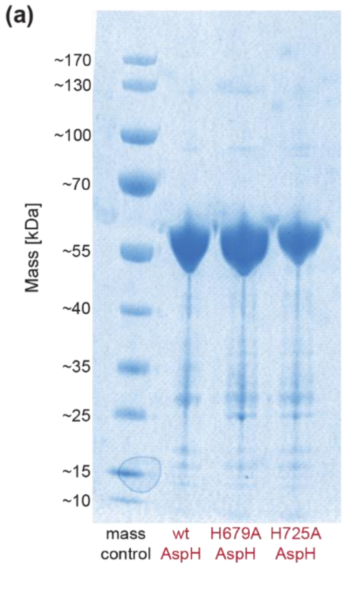

Deconvoluted mass spectra of (**b**, and a close-up in **c**) the purified H679A His$_6$-AspH$_{315-758}$ variant and (**d**, and a close-up in **e**) the purified H725A His$_6$-AspH$_{315-758}$ variant after size-exclusion chromatography show the expected calculated mass (in red) of the His$_6$-AspH$_{315-758}$ variants (H679A His$_6$-AspH$_{315-758}$: 54.452 kDa, calculated 54.453 kDa; H725A His$_6$-AspH$_{315-758}$: 54.452 kDa, calculated 54.453 kDa) and an additional species (+ 178 Da, in blue) corresponding to His$_6$-AspH$_{315-758}$ variants containing a gluconoylated His$_6$-tag, a common covalent modification of recombinant *N*-terminal His$_6$-tagged proteins produced in *E. coli*[2], which does not impact substantially (if at all) on AspH-catalysis.

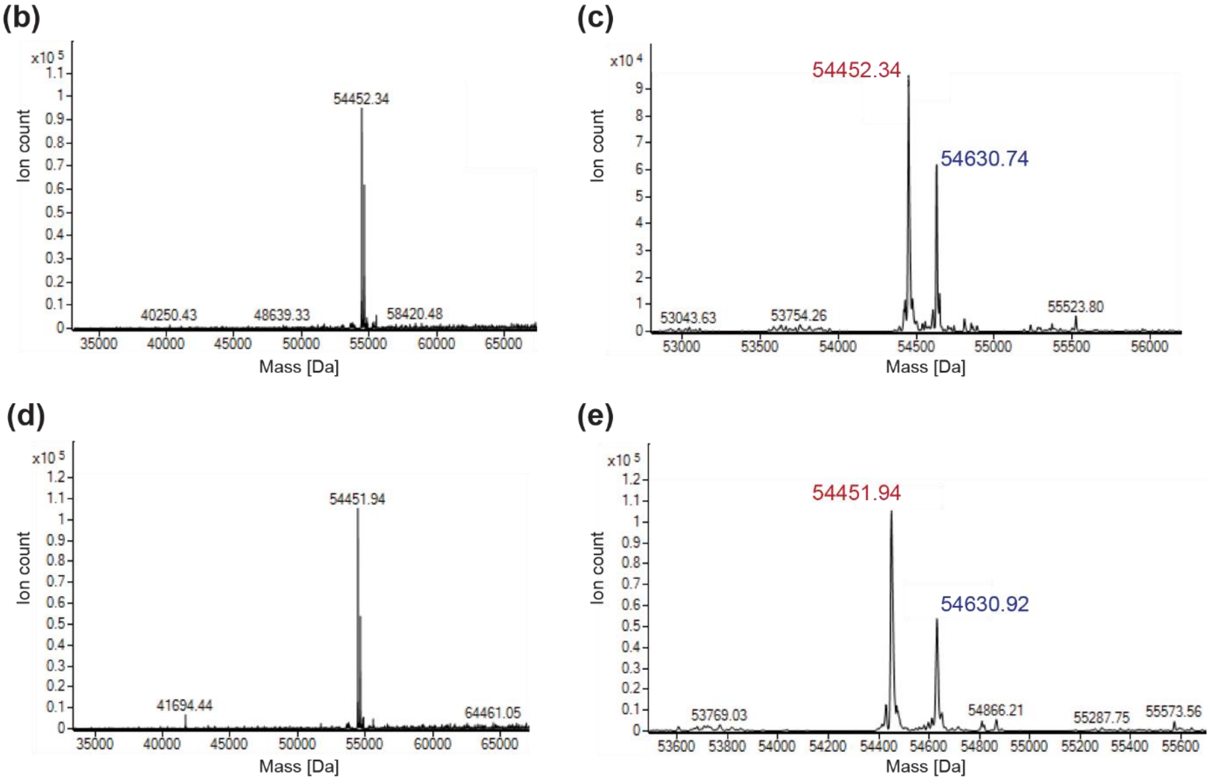

Supporting Figure S2. Initial turnover assays of wt AspH and its H679A and H725A variants reveal catalytic activity. (**a**) The hFX-EGFD1$_{86\text{-}124}$-4Ser peptide[1] used for kinetic and crystallographic experiments is based on the sequence of EGFD1 of the confirmed AspH substrate human coagulation Factor X (hFX).[3] The peptide macrocycle formed by the disulphide between Cys101$_{hFX}$ and Cys110$_{hFX}$ mimics a non-canonical (*i.e.* Cys 1–2, 3–4, 5–6) EGFD disulfide pattern, which has been previously identified as a substrate requirement for AspH catalysis.[1] The peptide was synthesized and purified by GL Biochem (Shanghai) Ltd. The AspH hydroxylation site (Asp103$_{hFX}$) is in red; cystine sulfurs are in green; serine residues substituting for hFX cysteine residues are in light blue; numbering is according to the sequence of hFX;[3] (**b**) under the reaction conditions, wt His$_6$-AspH$_{315\text{-}758}$ catalyses the hydroxylation of the hFX-EGFD1$_{86\text{-}124}$-4Ser peptide substantially faster than the H679A variant (>95% conversion after 30 min vs ~50% conversion for H679A AspH), which is much more active than the H725A variant (<5% conversion after 30 min). Reactions were initiated by addition of the His$_6$-AspH$_{315\text{-}758}$ variants (final concentration: 0.3 µM) to the reaction mixture containing the hFX-EGFD1$_{86\text{-}124}$-4Ser peptide (4.0 µM), 2OG (200 µM), LAA (400 µM), and FAS (10 µM) in reaction buffer (50 mM Tris, 50 mM NaCl, pH 7.5) in separate wells of a 96 deep well plate (Greiner). The AspH-catalysed hydroxylation of the hFX-EGFD1$_{86\text{-}124}$-4Ser peptide was monitored by SPE-MS.

(a)

hFX EGFD1$_{86\text{-}124}$-4Ser (hFX amino acids 86-124; Cys101$_{hFX}$- and Cys110$_{hFX}$-thiols form a disulfide bond):

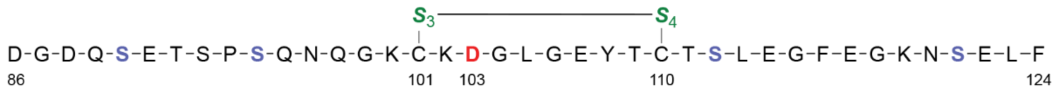

(b)

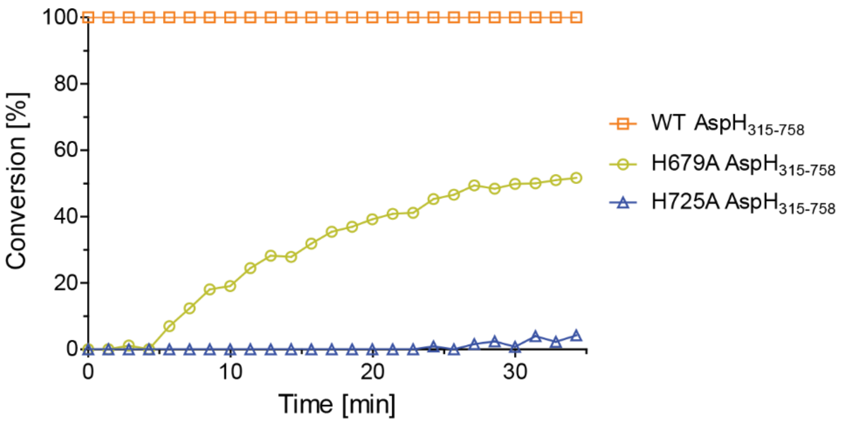

Supporting Figure S3. The catalytic activity of the AspH variants was highest in HEPES buffer in the absence of NaCl. H679A His$_6$-AspH$_{315-758}$ activity decreases with increasing NaCl concentration and increasing pH, in agreement with a study on wt AspH.[4] The H679A AspH variant was more active in 50 mM HEPES buffer (pH 7.5) than in 50 mM Tris buffer (pH 7.5). The HEPES concentration was reduced to 25 mM in order to improve peptide detection by MS. Optimized buffer conditions for all subsequent kinetic and inhibition SPE-MS assays of the H679A and H725A AspH variants were: 25 mM HEPES, pH 7.5. Reactions were initiated by addition of the H679A His$_6$-AspH$_{315-758}$ variant (final concentration: 0.3 μM) to the reaction mixture containing the hFX-EGFD1$_{86-124}$-4Ser peptide (4.0 μM), 2OG (200 μM), LAA (400 μM), and FAS (10 μM) in reaction buffer. AspH-catalysed hydroxylation of the hFX-EGFD1$_{86-124}$-4Ser peptide (Supporting Figure S2a) was monitored by SPE-MS.

(**a**) Buffer: 50 mM Tris, pH 7.5. Colour code: orange boxes: 0 mM NaCl, yellow circles: 25 mM NaCl, blue triangle: 50 mM NaCl, violet diamond: 100 mM NaCl; (**b**) buffer: 50 mM Tris, pH 8.0. Colour code: orange boxes: 0 mM NaCl, yellow circles: 25 mM NaCl, blue triangle: 50 mM NaCl, violet diamond: 100 mM NaCl; (**c**) buffer: 50 mM HEPES, pH 7.5. Colour code: orange boxes: 0 mM NaCl, blue triangle: 50 mM NaCl, violet diamond: 100 mM NaCl; (**d**) direct comparison of HEPES and Tris reaction buffers using 0.3 μM H679A AspH (enzyme/substrate ratio = 1/20). Colour code: green boxes: 50 mM HEPES (pH 7.5, 0 mM NaCl), red circles: 50 mM Tris (pH 7.5, 0 mM NaCl).

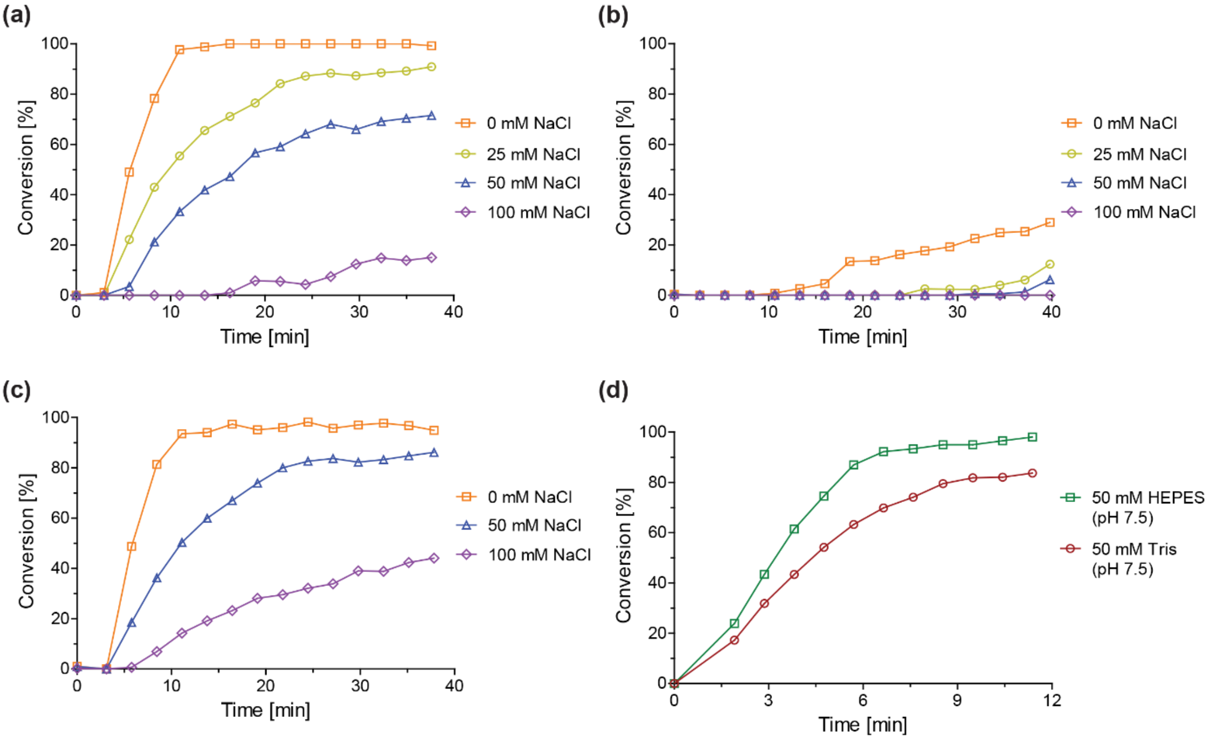

Supporting Figure S4. Kinetic parameters for wt AspH (continues on the following page). Turnover numbers (k_{cat}^{app}) and Michaelis constants (K_m^{app}) of wt AspH were determined in independent triplicates for 2OG and ammonium iron(II) sulfate hexahydrate (FAS, $(NH_4)_2Fe(SO_4)_2 \cdot 6H_2O$), both in the presence and absence of L-ascorbic acid (LAA) monitoring the AspH-catalysed hydroxylation of the hFX-EGFD1$_{86-124}$-4Ser peptide (Supporting Figure S2a) by SPE-MS (SPE-MS parameters are given in Section 2 of the Supporting Information). Note that the hFX-EGFD1$_{86-124}$-4Ser peptide substrate cannot be detected by SPE-MS at low concentrations, hampering an accurate determination of its wt AspH K_m-value. Conditions: 0.1 µM wt His$_6$-AspH$_{315-758}$ using variable concentrations of 2OG, FAS, and hFX-EGFD1$_{86-124}$-4Ser, respectively, in 25 mM HEPES (pH 7.5, 20° C; note, to increase enzyme activity, 50 mM NaCl was added to the reaction buffer). Measurement times were normalized to the first sample injection analysed after the addition of the Enzyme Mixture to the Substrate Mixture (t = 0 s), by which time low levels of hydroxylation were manifest. Data are shown as the mean average of three independent runs (n = 3; mean ± standard deviation, SD). Turnover numbers (k_{cat}^{app}) were calculated from the maximum velocities (v_{max}^{app}) values obtained, assuming ideal active site availability as supported by a reported active site titration of wt AspH.[4]

(**a**) Time course of the wt AspH-catalysed hydroxylation reaction of the hFX-EGFD1$_{86-124}$-4Ser peptide substrate in the absence of LAA for the shown Fe(II) concentrations (conditions: 0.1 µM wt His$_6$-AspH$_{315-758}$, 20 µM 2OG, and 4.0 µM hFX-EGFD1$_{86-124}$-4Ser); (**b**) hydroxylation rates used to determine kinetic parameters of wt AspH for Fe(II) in the absence of LAA; (**c**) K_m^{app} of wt AspH for Fe(II) in the absence of LAA.

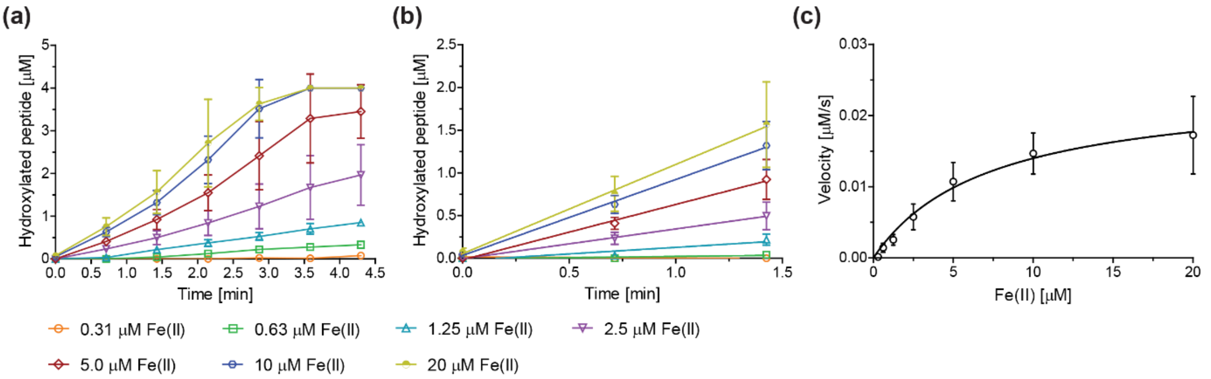

(**d**) Time course of the wt AspH-catalysed hydroxylation reaction of the hFX-EGFD1$_{86-124}$-4Ser peptide substrate in the presence of LAA for the shown Fe(II) concentrations (conditions: 0.1 µM wt His$_6$-AspH$_{315-758}$, 100 µM LAA, 20 µM 2OG, and 4.0 µM hFX-EGFD1$_{86-124}$-4Ser); (**e**) hydroxylation rates used to determine kinetic parameters of wt AspH for Fe(II) in the presence of LAA; (**f**) K_m^{app} of wt AspH for Fe(II) in the presence of LAA.

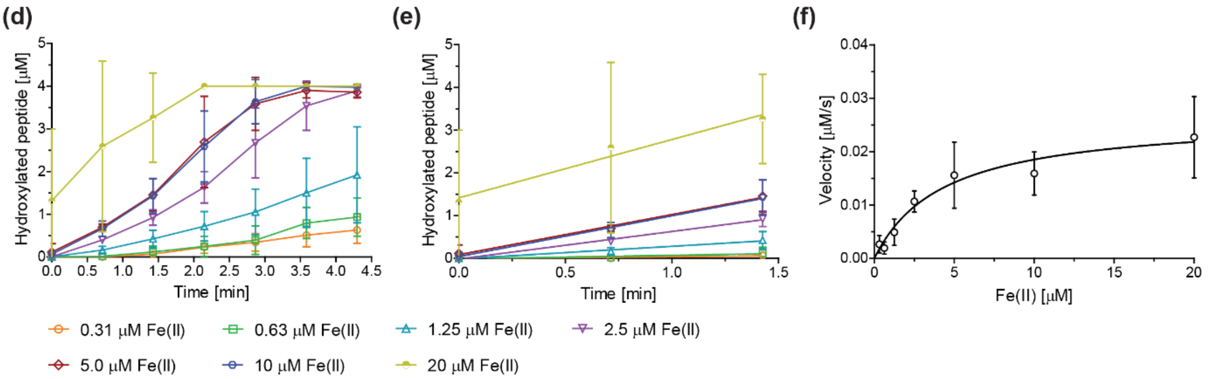

(**g**) Time course of the wt AspH-catalysed hydroxylation reaction of the hFX-EGFD1$_{86\text{-}124}$-4Ser peptide substrate for the shown 2OG concentrations (conditions: 0.1 µM wt His$_6$-AspH$_{315\text{-}758}$, 100 µM LAA, 20 µM Fe(II), and 4.0 µM hFX-EGFD1$_{86\text{-}124}$-4Ser); (**h**) hydroxylation rates used to determine kinetic parameters of wt AspH for 2OG; (**i**) K_m^{app} of wt AspH for 2OG.

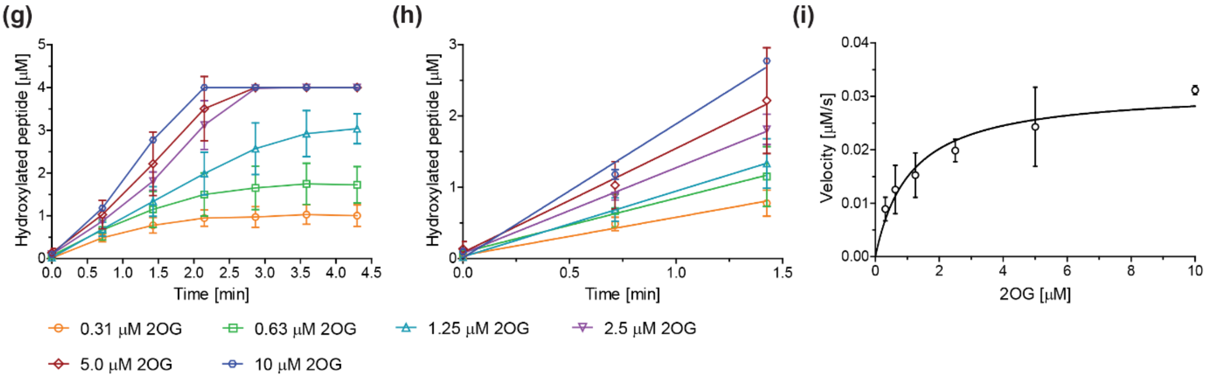

Supporting Figure S5. Kinetic parameters for H679A AspH (continues on the following page). Turnover numbers (k_{cat}^{app} or k_{cat}) and Michaelis constants (K_m^{app} or K_m) of the H679A AspH variant were determined in independent triplicates for 2OG, ammonium iron(II) sulfate hexahydrate (FAS, $(NH_4)_2Fe(SO_4)_2 \cdot 6H_2O$), both in the presence and absence of L-ascorbic acid (LAA), and the hFX-EGFD1$_{86\text{-}124}$-4Ser peptide substrate (Supporting Figure S2a), monitoring the H679A AspH-catalysed hydroxylation of the hFX-EGFD1$_{86\text{-}124}$-4Ser peptide by SPE-MS (SPE-MS parameters are given in Section 2 of the Supporting Information). Conditions: 0.2 µM H679A His$_6$-AspH$_{315\text{-}758}$ using variable concentrations of 2OG, FAS, and hFX-EGFD1$_{86\text{-}124}$-4Ser, respectively, in 25 mM HEPES (pH 7.5, 20° C). Measurement times were normalized to the first sample injection analysed after the addition of the Enzyme Mixture to the Substrate Mixture (t = 0 s), by which time low levels of hydroxylation were manifest. Data are shown as the mean average of three independent runs (n = 3; mean ± standard deviation, SD). Turnover numbers (k_{cat}^{app} or k_{cat}) were calculated from the maximum velocities (v_{max}^{app} or v_{max}) values obtained, assuming ideal active site availability as supported by a reported active site titration of wt AspH.[4]

(**a**) Time course of the H679A AspH-catalysed hydroxylation reaction of the hFX-EGFD1$_{86\text{-}124}$-4Ser peptide substrate in the absence of LAA for the shown Fe(II) concentrations (conditions: 0.2 µM H679A His$_6$-AspH$_{315\text{-}758}$, 2000 µM 2OG, and 4.0 µM hFX-EGFD1$_{86\text{-}124}$-4Ser); (**b**) hydroxylation rates used to determine kinetic parameters of H679A AspH for Fe(II) in the absence of LAA; (**c**) K_m^{app} of H679A AspH for Fe(II) in the absence of LAA.

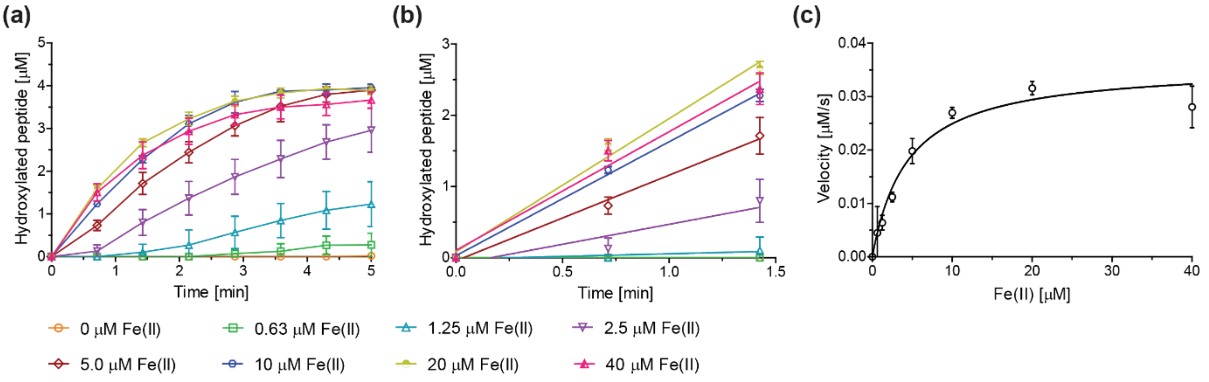

(**d**) Time course of the H679A AspH-catalysed hydroxylation reaction of the hFX-EGFD1$_{86\text{-}124}$-4Ser peptide substrate in the presence of LAA for the shown Fe(II) concentrations (conditions: 0.2 µM H679A His$_6$-AspH$_{315\text{-}758}$, 400 µM LAA, 2000 µM 2OG, and 4.0 µM hFX-EGFD1$_{86\text{-}124}$-4Ser); (**e**) hydroxylation rates used to determine kinetic parameters of H679A AspH for Fe(II) in the presence of LAA; (**f**) K_m^{app} of H679A AspH for Fe(II) in the presence of LAA.

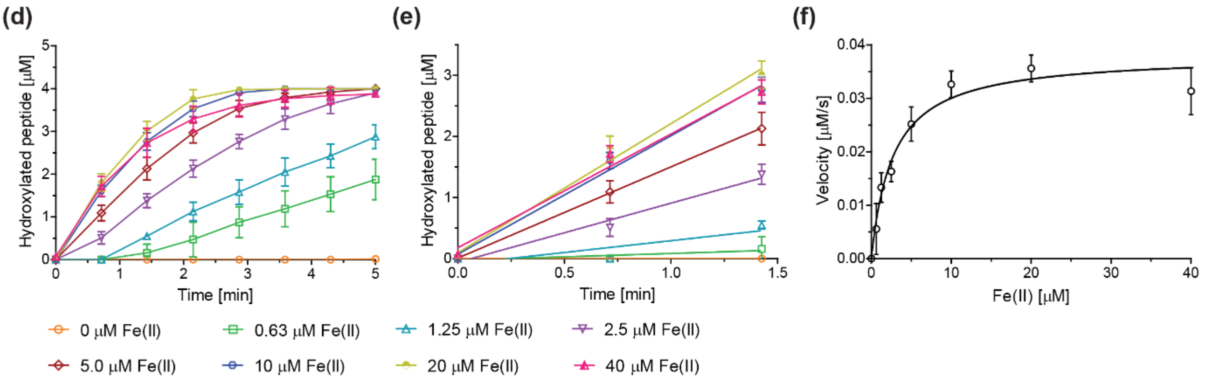

(**g**) Time course of the H679A AspH-catalysed hydroxylation reaction of the hFX-EGFD1$_{86\text{-}124}$-4Ser peptide substrate for the shown 2OG concentrations (conditions: 0.2 µM H679A His$_6$-AspH$_{315\text{-}758}$, 400 µM LAA, 20 µM Fe(II), and 4.0 µM hFX-EGFD1$_{86\text{-}124}$-4Ser); (**h**) hydroxylation rates used to determine kinetic parameters of H679A AspH for 2OG; (**i**) $K_\mathrm{m}^\mathrm{app}$ of H679A AspH for 2OG.

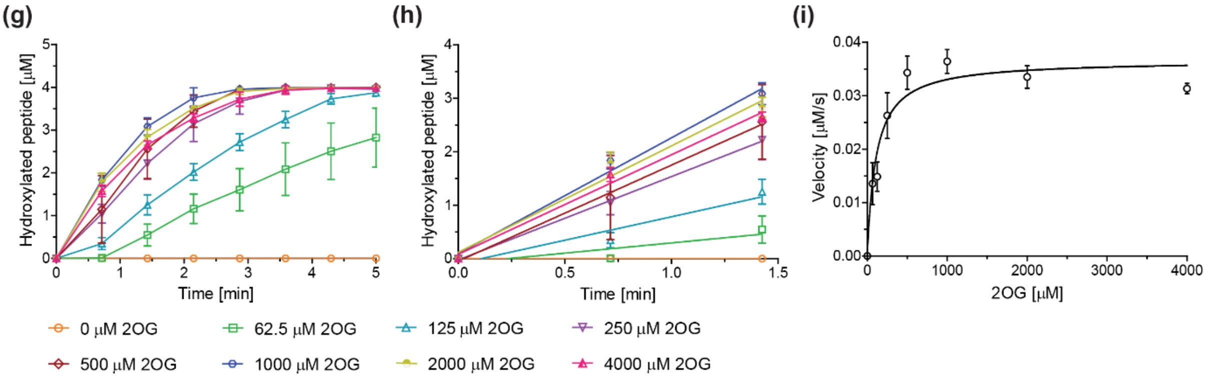

(**j**) Time course of the H679A AspH variant-catalysed hydroxylation reaction of the hFX-EGFD1$_{86\text{-}124}$-4Ser peptide substrate for the shown hFX-EGFD1$_{86\text{-}124}$-4Ser concentrations (conditions: 0.2 µM H679A His$_6$-AspH$_{315\text{-}758}$, 400 µM LAA, 2000 µM 2OG, and 20 µM FAS); (**k**) hydroxylation rates used to determine kinetic parameters of H679A AspH for hFX-EGFD1$_{86\text{-}124}$-4Ser. Note that the hFX-EGFD1$_{86\text{-}124}$-4Ser peptide substrate cannot be detected by SPE-MS at concentrations below 1.0 µM, hampering a more accurate determination of the K_m-value for hFX-EGFD1$_{86\text{-}124}$-4Ser; (**l**) K_m of H679A AspH for the hFX-EGFD1$_{86\text{-}124}$-4Ser peptide substrate.

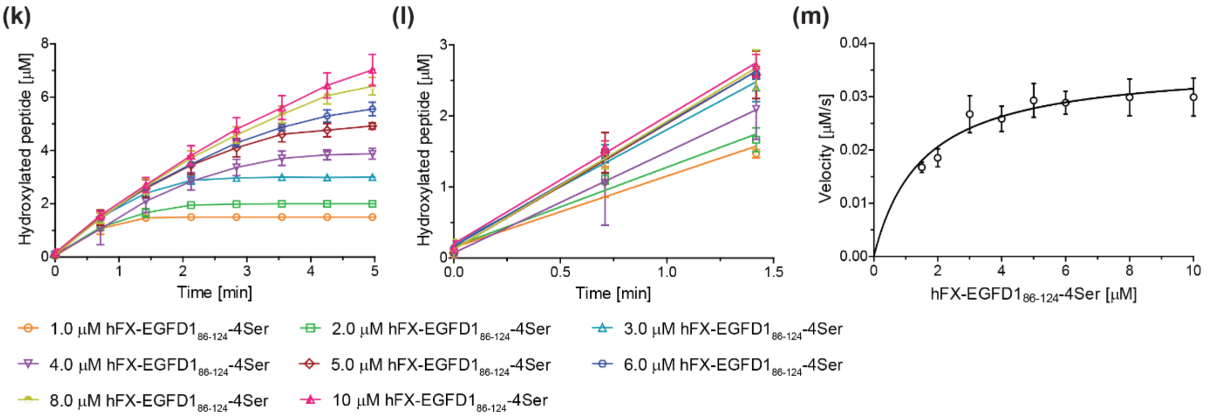

Supporting Figure S6. Kinetic parameters for H725A AspH (continues on the following page). Turnover numbers (k_{cat}^{app} or k_{cat}) and Michaelis constants (K_m^{app} or K_m) of the H725A AspH variant were determined in independent triplicates for 2OG, ammonium iron(II) sulfate hexahydrate (FAS, $(NH_4)_2Fe(SO_4)_2 \cdot 6H_2O$), both in the presence and absence of L-ascorbic acid (LAA), and the hFX-EGFD1$_{86\text{-}124}$-4Ser peptide substrate (Supporting Figure S2a), monitoring the H725A AspH-catalysed hydroxylation of the hFX-EGFD1$_{86\text{-}124}$-4Ser peptide by SPE-MS (SPE-MS parameters are given in Section 2 of the Supporting Information). Conditions: 0.2 µM H725A His$_6$-AspH$_{315\text{-}758}$ using variable concentrations of 2OG, FAS, and hFX-EGFD1$_{86\text{-}124}$-4Ser, respectively, in 25 mM HEPES (pH 7.5, 20° C). Measurement times were normalized to the first sample injection analysed after the addition of the Enzyme Mixture to the Substrate Mixture (t = 0 s), by which time low levels of hydroxylation were manifest. Data are shown as the mean average of three independent runs (n = 3; mean ± standard deviation, SD). Turnover numbers (k_{cat}^{app} or k_{cat}) were calculated from the maximum velocities (v_{max}^{app} or v_{max}) values obtained, assuming ideal active site availability as supported by a reported active site titration of wt AspH.[4]

(**a**) Time course of the H725A AspH-catalysed hydroxylation reaction of the hFX-EGFD1$_{86\text{-}124}$-4Ser peptide substrate in the absence of LAA for the shown Fe(II) concentrations (conditions: 0.2 µM H725A His$_6$-AspH$_{315\text{-}758}$, 2000 µM 2OG, and 4.0 µM hFX-EGFD1$_{86\text{-}124}$-4Ser); (**b**) hydroxylation rates used to determine kinetic parameters of H725A AspH for Fe(II) in the absence of LAA; (**c**) K_m^{app} of H725A AspH for Fe(II) in the absence of LAA.

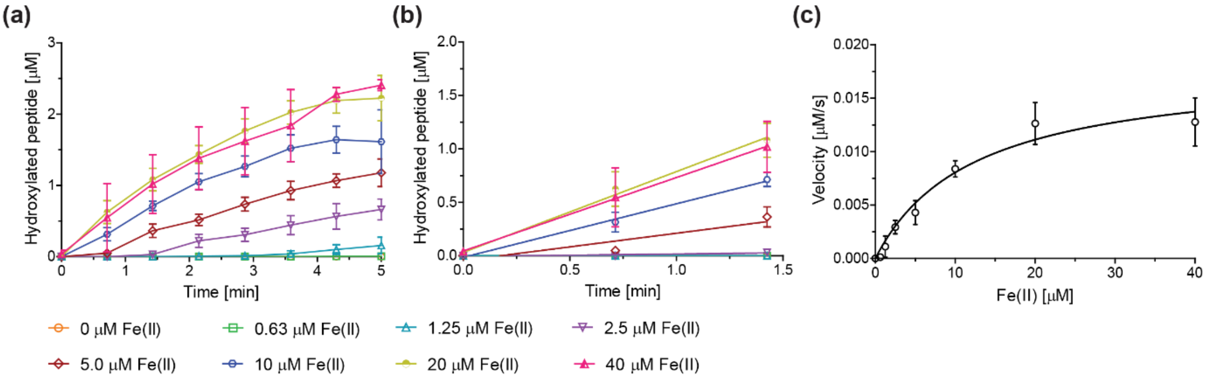

(**d**) Time course of the H725A AspH-catalysed hydroxylation reaction of the hFX-EGFD1$_{86\text{-}124}$-4Ser peptide substrate in the presence of LAA for the shown Fe(II) concentrations (conditions: 0.2 µM H725A His$_6$-AspH$_{315\text{-}758}$, 400 µM LAA, 2000 µM 2OG, and 4.0 µM hFX-EGFD1$_{86\text{-}124}$-4Ser); (**e**) hydroxylation rates used to determine kinetic parameters of H725A AspH for Fe(II) in the presence of LAA; (**f**) K_m^{app} of H725A AspH for Fe(II) in the presence of LAA.

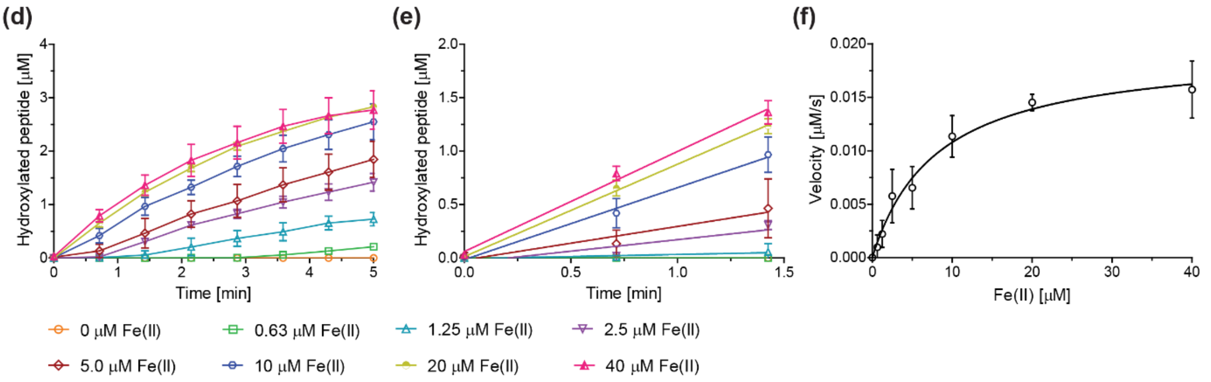

(**g**) Time course of the H725A AspH-catalysed hydroxylation reaction of the hFX-EGFD1$_{86\text{-}124}$-4Ser peptide substrate for the shown 2OG concentrations (conditions: 0.2 µM H725A His$_6$-AspH$_{315\text{-}758}$, 400 µM LAA, 20 µM Fe(II), and 4.0 µM hFX-EGFD1$_{86\text{-}124}$-4Ser); (**h**) hydroxylation rates used to determine kinetic parameters of H725A AspH for 2OG; (**i**) K_m^{app} of H725A AspH for 2OG.

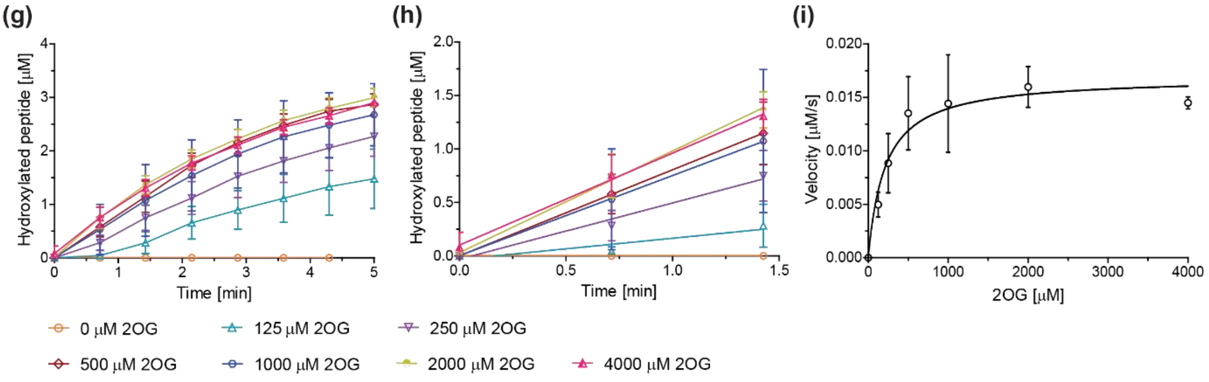

(**j**) Time course of the H725A AspH variant-catalysed hydroxylation reaction of the hFX-EGFD1$_{86\text{-}124}$-4Ser peptide substrate for the shown hFX-EGFD1$_{86\text{-}124}$-4Ser concentrations (conditions: 0.2 µM H725A His$_6$-AspH$_{315\text{-}758}$, 400 µM LAA, 2000 µM 2OG, and 20 µM FAS); (**k**) hydroxylation rates used to determine kinetic parameters of H725A AspH for hFX-EGFD1$_{86\text{-}124}$-4Ser. Note that the hFX-EGFD1$_{86\text{-}124}$-4Ser peptide substrate cannot be detected by SPE-MS at concentrations below 1.0 µM, hampering a more accurate determination of the K_m-value for hFX-EGFD1$_{86\text{-}124}$-4Ser; (**l**) K_m of H725A AspH for the hFX-EGFD1$_{86\text{-}124}$-4Ser peptide substrate.

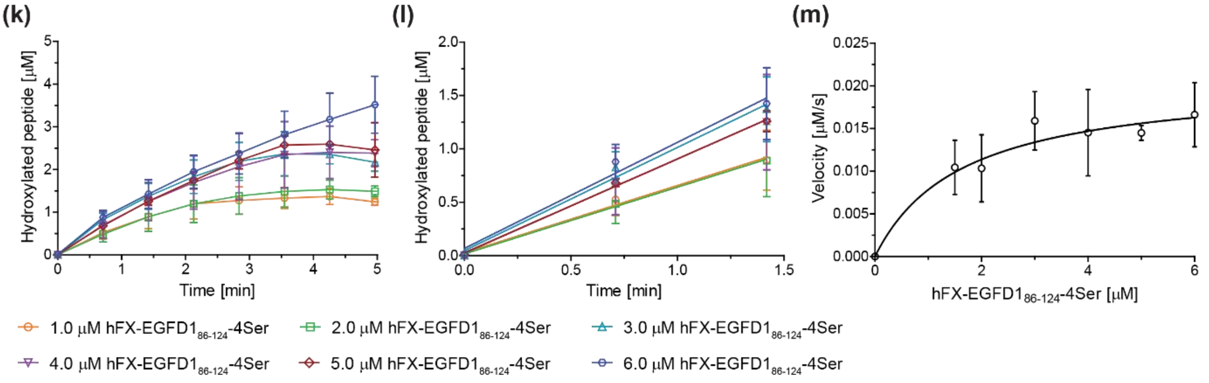

Supporting Figure S7. The H679A and H725A AspH variants do not require L-ascorbic acid for activity. The kinetic parameters of the H679A and H725A AspH variants were determined in independent triplicates for L-ascorbic acid (LAA), monitoring the AspH variant-catalysed hydroxylation of the hFX-EGFD1$_{86\text{-}124}$-4Ser peptide (Supporting Figure S2a) by SPE-MS (SPE-MS parameters are given in Section 2 of the Supporting Information). Conditions: 0.2 µM H679A or H725A His$_6$-AspH$_{315\text{-}758}$ in 25 mM HEPES (pH 7.5, 20° C), 4.0 µM hFX-EGFD1$_{86\text{-}124}$-4Ser, 2000 µM 2OG, 20 µM ammonium iron(II) sulfate hexahydrate (FAS), and variable concentrations of LAA. Measurement times were normalized to the first sample injection analysed after the addition of the Enzyme Mixture to the Substrate Mixture (t = 0 s), by which time low levels of hydroxylation were manifest. Data are shown as the mean average of three independent runs (n = 3; mean ± standard deviation, SD). From the initial maximum velocities (v_{max}^{app}) values obtained, turnover numbers (k_{cat}^{app}) were calculated assuming ideal active site availability as supported by an active site titration of wt AspH.[4]

(**a** and **b**) Time courses of the (**a**) H679A and (**b**) H725A AspH variant-catalysed hydroxylation reaction of the hFX-EGFD1$_{86\text{-}124}$-4Ser substrate peptide for the shown concentrations of LAA; (**c** and **d**) hydroxylation rates used to determine kinetic parameters of the (**c**) H679A and (**d**) H725A AspH variant for LAA; (**e** and **f**) effect of LAA on the (**e**) H679A and (**f**) H725A AspH variant activity, the AspH variant k_{cat}^{app}-values for LAA are constant throughout the concentration range investigated, including in the absence of LAA indicating that LAA is not required for the AspH variant activity. The AspH variant k_{cat}^{app}-values for LAA are in the range of those determined when varying the 2OG, Fe(II), or substrate peptide concentrations.

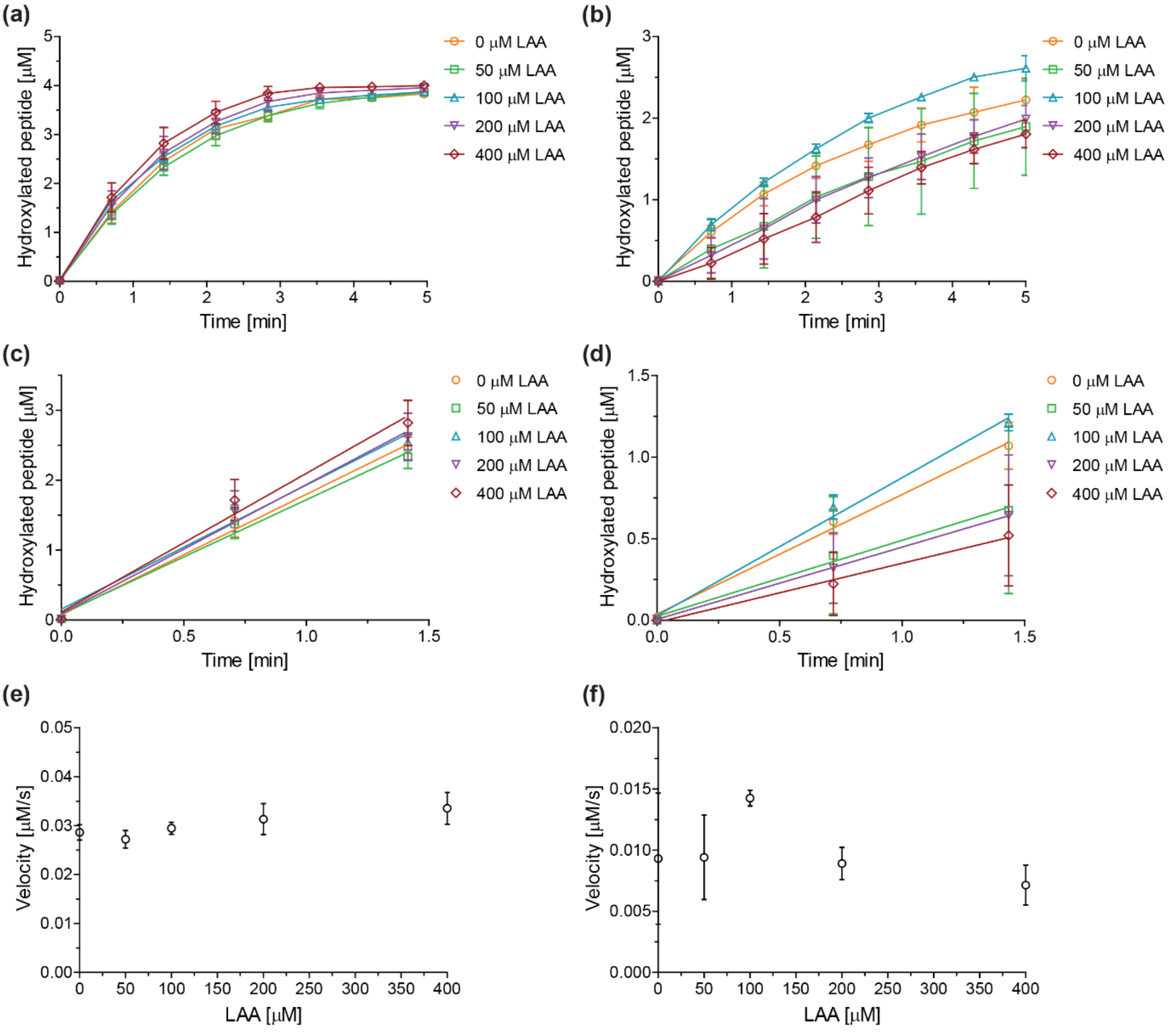

Supporting Figure S8. The H679A and H725A AspH variants do not efficiently catalyse the oxidative decarboxylation of 2OG to succinate and CO_2 in the absence of substrate (continues on the following page). The H679A and H725A AspH variant-catalysed oxidative decarboxylation of 2OG uncoupled from substrate oxidation was monitored using a Bruker AVIII 700 NMR machine equipped with a 5mm $^{1}H(^{13}C/^{15}N)$ inverse cryoprobe. Assay conditions: 1 µM H679A or H725A His_6-$AspH_{315-758}$ variant, 200 µM 2OG, and 20 µM ammonium iron(II) sulfate hexahydrate (FAS, $(NH_4)_2Fe(SO_4)_2 \cdot 6H_2O$) in 50 mM aqueous Tris-$d_{11}$ containing $10\%_{v/v}$ D_2O (pH 7.5, 25° C).

(I) Using H679A His_6-$AspH_{315-758}$: (**a**) Close-up (3.0-2.2 ppm) of the ^{1}H NMR spectrum of commercially sourced 2OG in 50 mM aqueous Tris-d_{11} containing $10\%_{v/v}$ D_2O (pH 7.5, 25° C); (**b**) close-up (3.0-2.2 ppm) of the ^{1}H NMR spectrum of commercially sourced succinate in 50 mM aqueous Tris-d_{11} containing $10\%_{v/v}$ D_2O (pH 7.5, 25° C); (**c** and **d**) a timecourse NMR experiment (t = 5 min, **c**; t = 60 min, **d**) reveals that the H679A AspH does not efficiently catalyse oxidative decarboxylation of 2OG to succinate and CO_2 in the absence of substrate. The results are in agreement NMR conversion assays using wt AspH.[1]

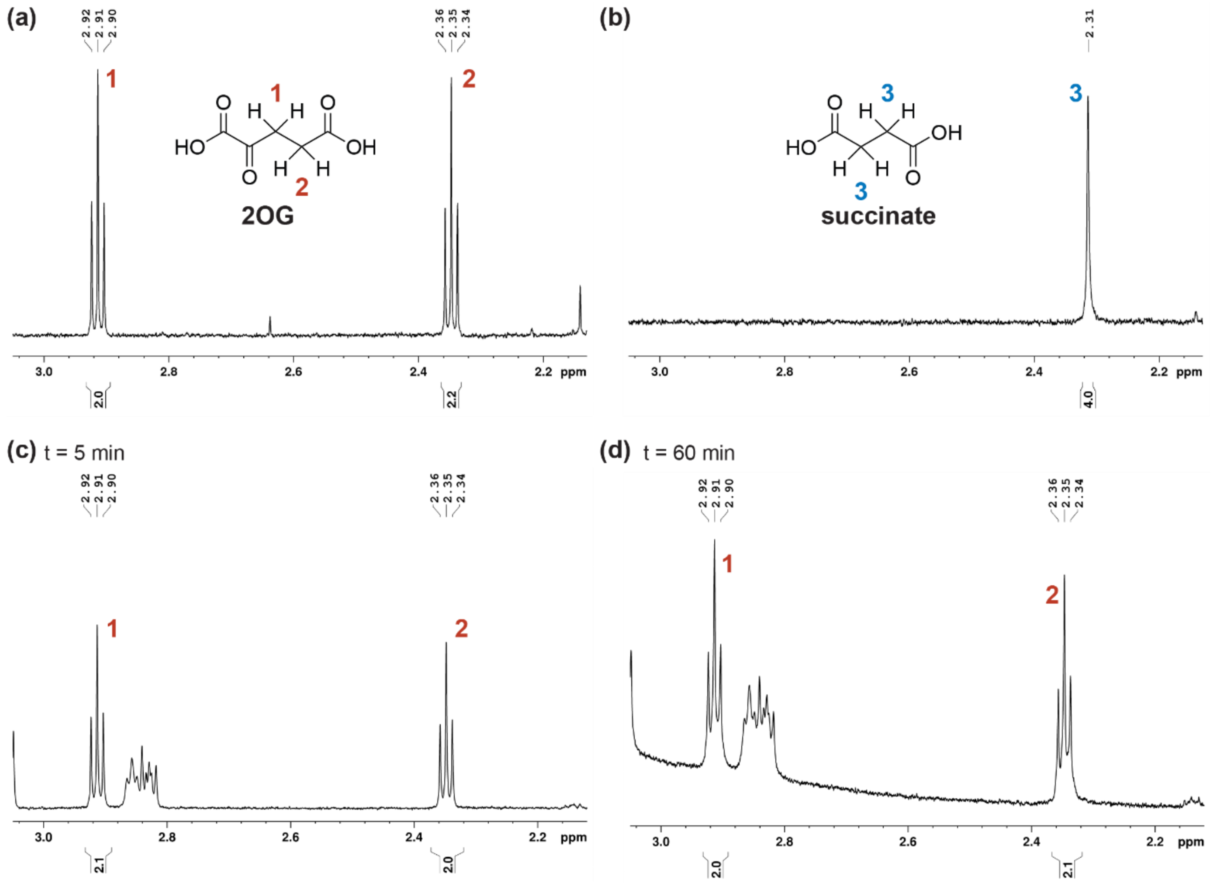

(II) Using H725A His_6-$AspH_{315-758}$: (**a**) Close-up (3.0-2.2 ppm) of the ^1H NMR spectrum of commercially sourced 2OG in 50 mM aqueous Tris-d_{11} containing $10\%_{v/v}$ D_2O (pH 7.5, 25° C); (**b**) close-up (3.0-2.2 ppm) of the ^1H NMR spectrum of commercially sourced succinate in 50 mM aqueous Tris-d_{11} containing $10\%_{v/v}$ D_2O (pH 7.5, 25° C); (**c** and **d**) a timecourse NMR experiment (t = 5 min, **c**; t = 60 min, **d**) reveals that the H725A AspH does not efficiently catalyse oxidative decarboxylation of 2OG to succinate and CO_2 in the absence of substrate. The results are in agreement NMR conversion assays using wt AspH.[1]

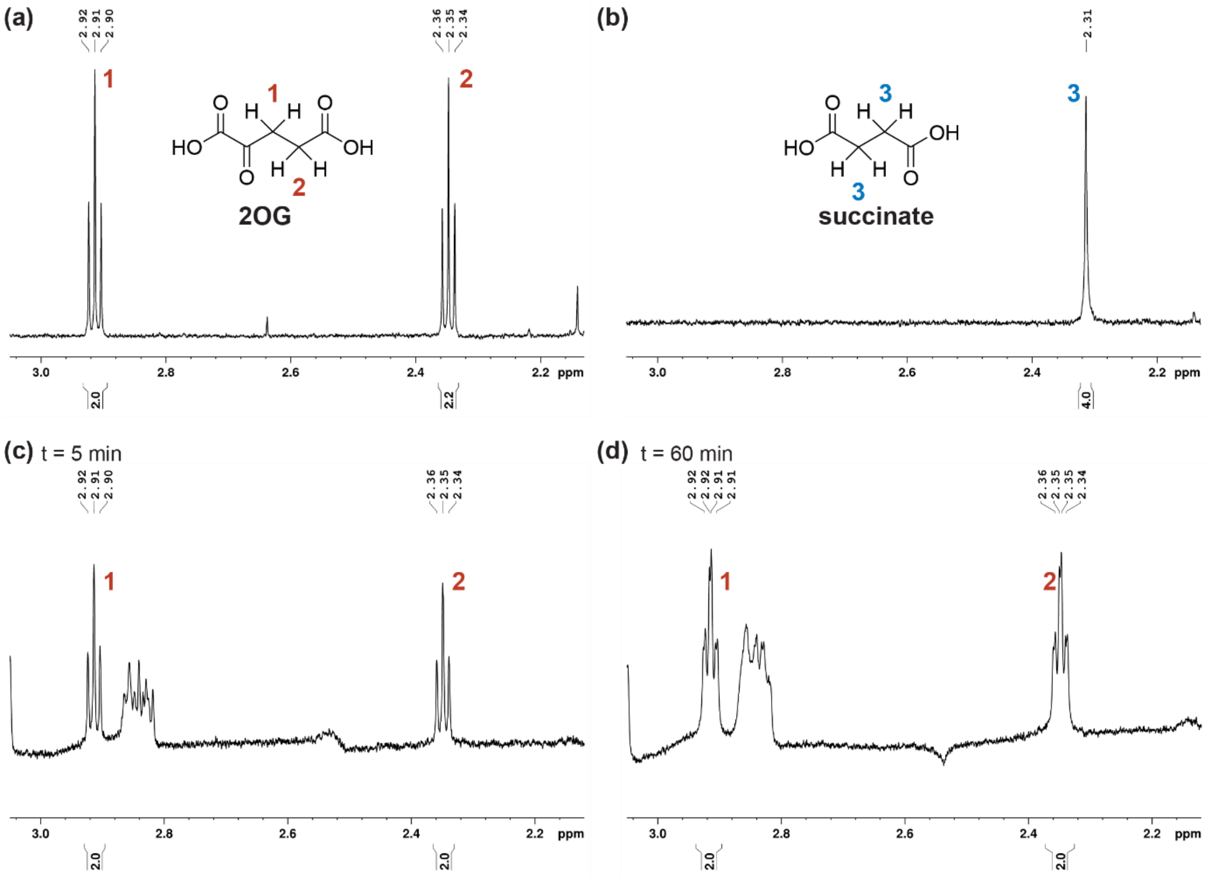

Supporting Figure S9. Robustness of the H679A and H725A AspH variant SPE-MS inhibition assays. SPE-MS inhibition assays were performed as described in Section 2 of the Supporting Information. (**a**) Z'-factors[5] and (**b**) signal-to-noise ratios (S/N) for the inhibition assay plates analysed to determine IC_{50}-values. The Z'-factors >0.5 (grey line) indicate a stable and robust assay of high quality.[5] Z'-factors and S/N-ratios were determined according to the literature using Microsoft Excel.[5] Colour code: H679A AspH variant: orange circles; H725A AspH variant: green boxes.

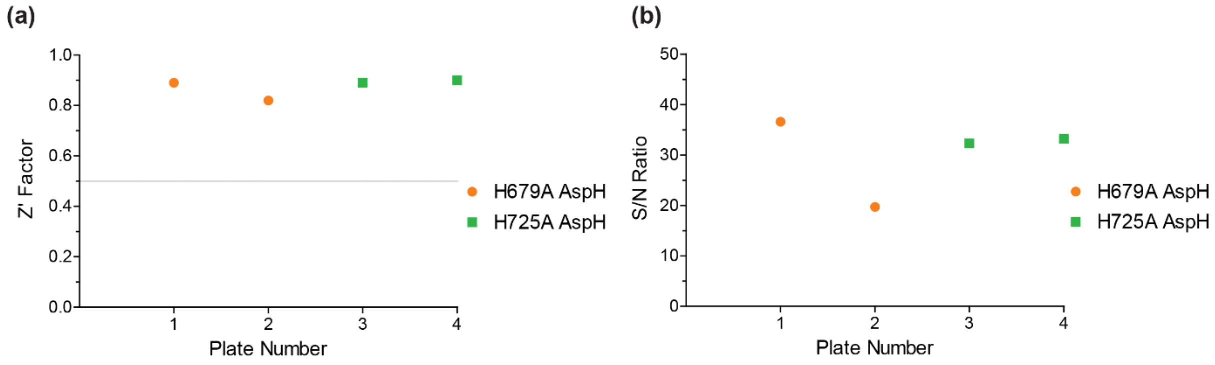

Supporting Figure S10. View of the protein fold from a crystal structure of H679A AspH with acetate present at the active site (H679A AspH:acetate; PDB ID: 6QA5). Overview of the H679A AspH:acetate crystal structure (2.7 Å resolution); Note, electron density for a metal ion bound to the H679A AspH active site was not observed. Colours: grey: H679A His_6-$AspH_{315-758}$; green: carbon-backbone of acetate; red: oxygen; blue: nitrogen. Note, electron density for a metal ion bound to the H679A AspH active site was not observed.

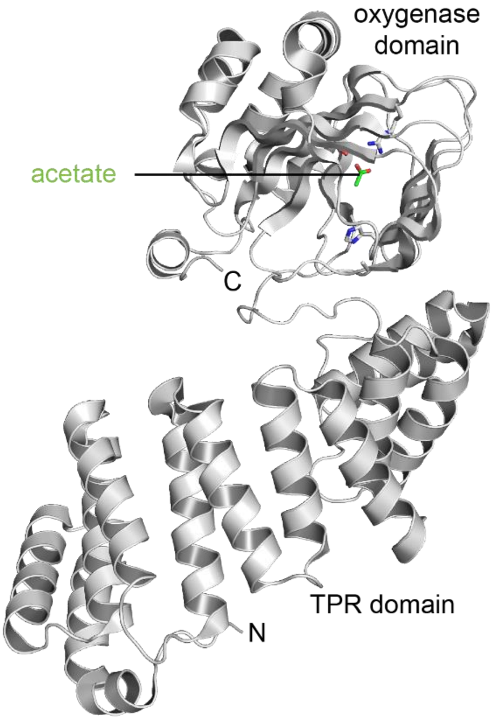

Supporting Figure S11. The substitution of H679 by alanine neither substantially alters the overall AspH fold nor the conformations of key active site residues. Colours: grey: H679A His$_6$-AspH$_{315\text{-}758}$; green: carbon-backbone of acetate; red: oxygen; blue: nitrogen. w: water.

(**a**) Superimposition of a view from the H679A AspH:acetate structure (Supporting Figure S10) with one from the reported AspH:Mn:NOG structure (bronze: wt AspH; yellow: carbon-backbone of NOG; violet: Mn; PDB ID: 5JZA)[1] reveals similar AspH conformations (Cα RMSD = 0.276 Å); (**b**) superimposition of a view from the active site of the H679A AspH:acetate structure with one from the reported AspH:Mn:NOG structure (bronze: wt AspH; yellow: carbon-backbone of NOG; violet: Mn; PDB ID: 5JZA)[1] reveals that the conformations of those wt AspH residues directly engaged in NOG/2OG binding (*i.e.* S668, R688, H690, and R735)[1,6] do not alter substantially upon substituting H679 for alanine, even though electron density for a metal ion is not observed. The conformation of H725, whose side chain imidazole engages in metal binding, does not alter substantially upon substituting H679 by alanine. Note, that no substantial conformational changes in the wt AspH structure have been reported to occur when substituting the broad-spectrum 2OG oxygenase inhibitor NOG by the natural cosubstrate 2OG.[6]

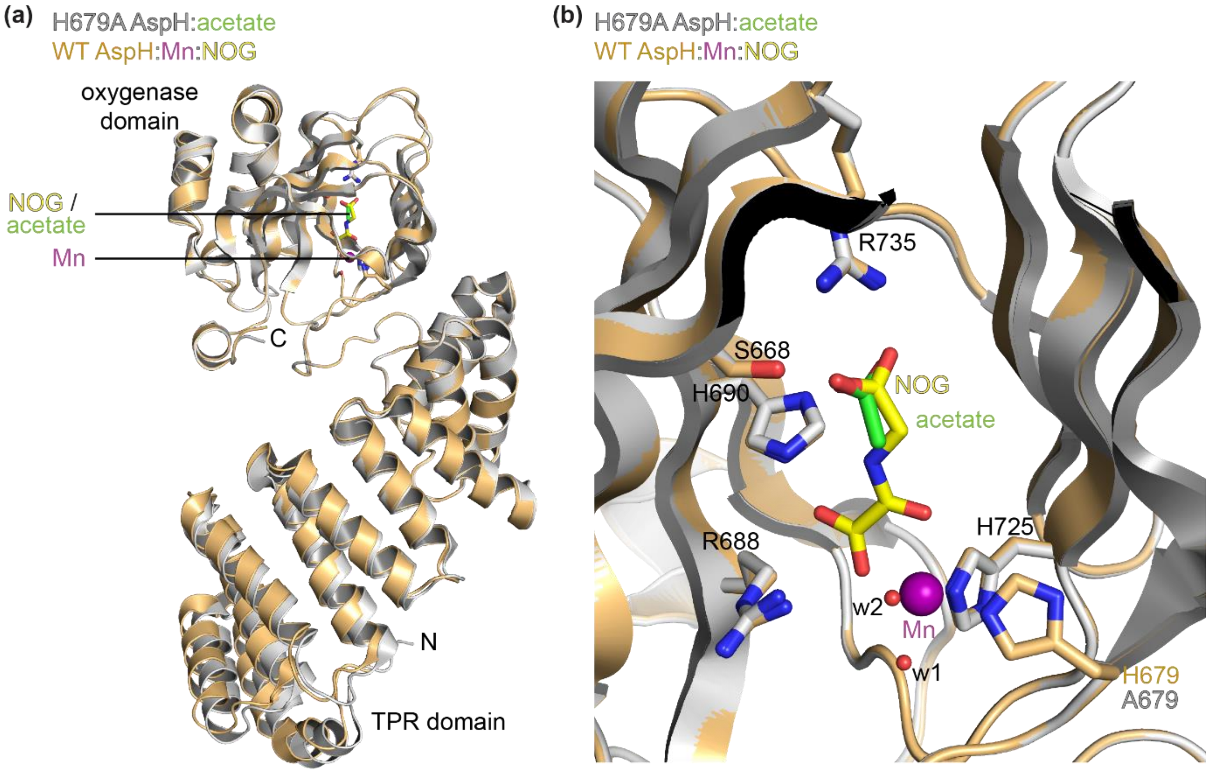

Supporting Figure S12. Views from a crystal structure of H679A AspH complexed with formate at the active site and a synthetic EGFD substrate peptide (H679A AspH:hFX-EGFD1$_{86\text{-}124}$-4Ser; PDB ID: 6Q9I). Colours: grey: H679A His$_6$-AspH$_{315\text{-}758}$; orange: carbon-backbone of formate; magenta: carbon-backbone of the hFX-EGFD1$_{86\text{-}124}$-4Ser peptide (Supporting Figure S2a); red: oxygen; blue: nitrogen.

(**a**) Overview of the H679A AspH:hFX-EGFD1$_{86\text{-}124}$-4Ser crystal structure (1.9 Å resolution). Note, electron density for a metal ion bound to the H679A AspH active site was not observed; (**b**) OMIT electron density map (mF_o-DF_c) contoured to 3σ around the hFX-EGFD1$_{86\text{-}124}$-4Ser peptide from the H679A AspH:hFX-EGFD1$_{86\text{-}124}$-4Ser structure reveals electron density for residues Gly99$_{hFX}$ to Ser112$_{hFX}$ including for the disulfide bridged (Cys101$_{hFX}$ and Cys110$_{hFX}$) ten-membered non-canonical EGFD macrocycle. Clear electron density is observed for the side chain of Tyr108$_{hFX}$, an EGFD residue which is part of the AspH-substrate consensus sequence requirement; the Tyr108$_{hFX}$ side chain interacts with the TPR domain of AspH as revealed in reported wt AspH:substrate complex structures.[1]

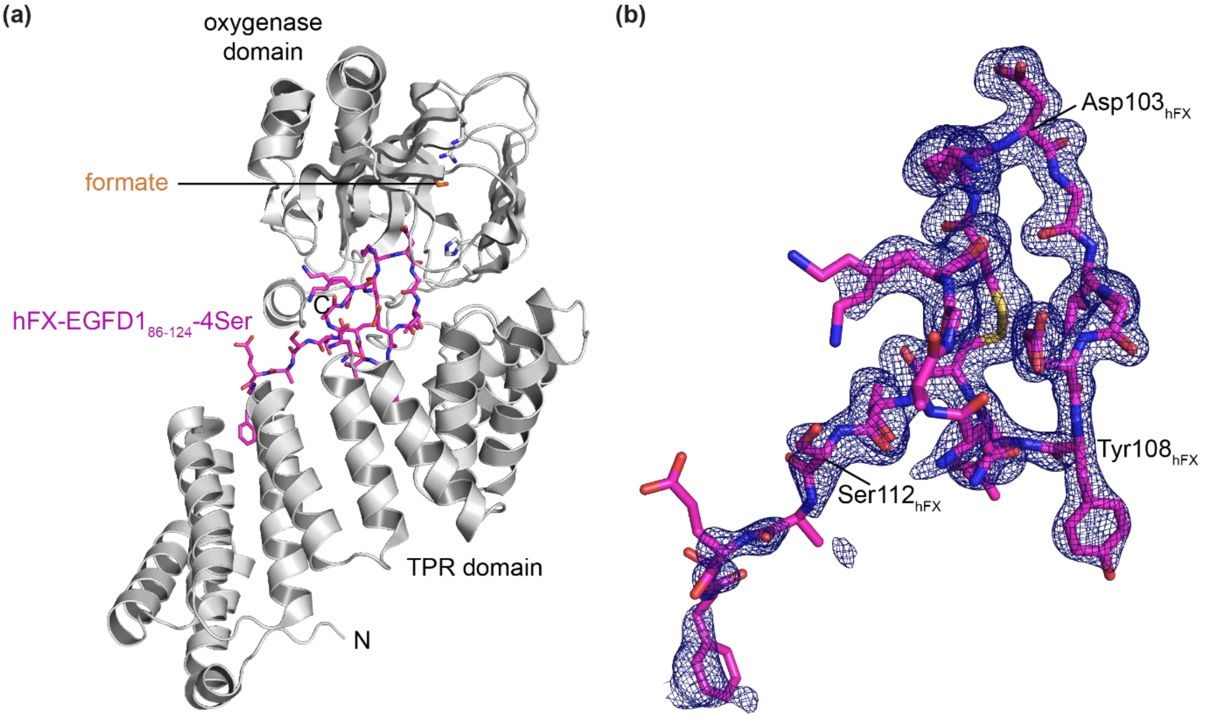

Supporting Figure S13. H679A AspH binds EGFD substrates through an induced fit mechanism in the absence of 2OG and metal ions. Colours: grey: H679A His$_6$-AspH$_{315\text{-}758}$; orange: carbon-backbone of formate; magenta: carbon-backbone of the hFX-EGFD1$_{86\text{-}124}$-4Ser peptide (Supporting Figure S2a); red: oxygen; blue: nitrogen.

(**a**) Superimposition of a view from the H679A AspH:hFX-EGFD1$_{86\text{-}124}$-4Ser structure (Supporting Figure S12) with one from the H679A AspH:acetate structure (H679A AspH: pale green; carbon-backbone of acetate: green; Supporting Figure S10) reveals that the distance between the Cα atoms of Leu433 on TPR repeat α6 and Pro756 in the H679A AspH C-terminal region decreases from ~18 Å to ~13 Å upon substrate binding (Cα RMSD = 2.755 Å). Evidence for an induced fit mechanism involving this conformational change has been described for wt AspH crystallized in the presence/absence of EGFD peptide substrates using NOG as an inhibitor or 2OG as a cosubstrate and Mn as metal ion substituting for Fe(II);[1,6] (**b**) superimposition of a view from the active site of the H679A AspH:hFX-EGFD1$_{86\text{-}124}$-4Ser structure (Supporting Figure S12) with one from the H679A AspH:acetate structure (H679A AspH: pale green; carbon-backbone of acetate: green; Supporting Figure S10) reveals that the conformations of those H679A AspH residues directly engaged in NOG/2OG binding in wt AspH (*i.e.* S668, R688, H690, and R735)[1,6] do not alter substantially upon substrate binding, even though electron density for a metal ion is not observed. The conformation of H725, whose side chain imidazole engages in metal binding in wt AspH, does not alter substantially upon substrate binding. The conformations of several (*i.e.* Glu615, Asp616, Glu617, Arg620, Gln627, and Gln632), but not all (*i.e.* Arg686 and Arg688), H679A AspH residues engaged in EGFD substrate binding change upon substrate binding in a similar manner as reported for wt AspH structures in the presence/absence of EGFD peptide substrates when using NOG as an inhibitor or 2OG as a cosubstrate and Mn as metal ion substituting for Fe(II).[1,6]

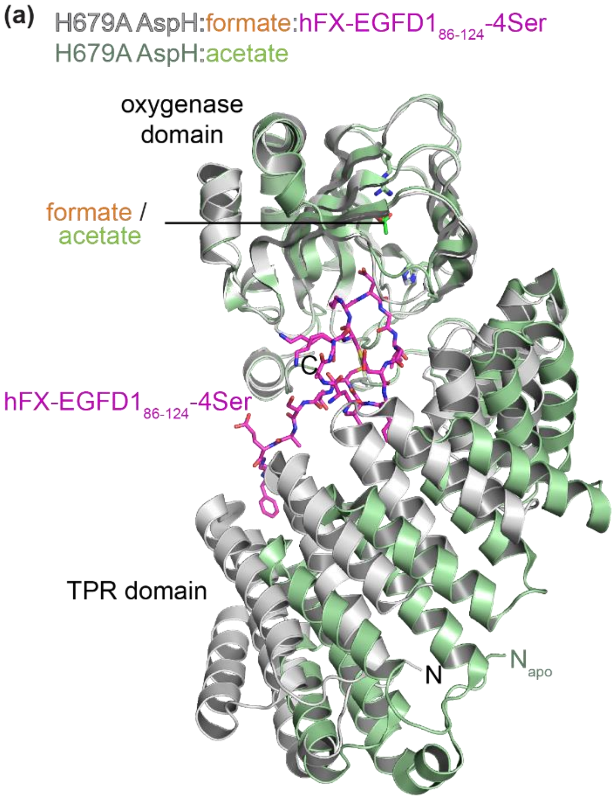

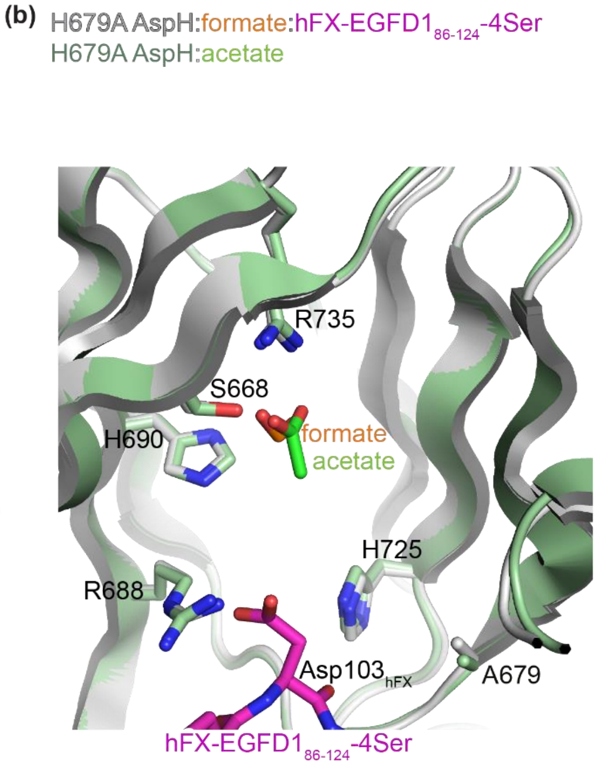

Supporting Figure S14. The substitution of H679 by alanine neither substantially alters the overall AspH fold nor the conformation of key active site residues in the presence of a substrate peptide. Colours: grey: H679A His$_6$-AspH$_{315-758}$; orange: carbon-backbone of formate; magenta: carbon-backbone of the hFX-EGFD1$_{86-124}$-4Ser peptide (Supporting Figure S2a); red: oxygen; blue: nitrogen. w: water.

(**a**) Superimposition of a view from the H679A AspH:hFX-EGFD1$_{86-124}$-4Ser structure (Supporting Figure S12) with one from the reported AspH:Mn:NOG:hFX-EGFD1$_{86-124}$-4Ser structure (bronze: wt AspH; yellow: carbon-backbone of NOG; cyan: carbon-backbone of the hFX-EGFD1$_{86-124}$-4Ser peptide; violet: Mn; PDB ID: 5JQY)[1] reveals similar AspH conformations (Cα RMSD = 0.300 Å); (**b**) superimposition of a view from the active site of the H679A AspH:hFX-EGFD1$_{86-124}$-4Ser structure with one from the reported AspH:Mn:NOG:hFX-EGFD1$_{86-124}$-4Ser structure (bronze: wt AspH; yellow: carbon-backbone of NOG; cyan: carbon-backbone of the hFX-EGFD1$_{86-124}$-4Ser peptide; violet: Mn; PDB ID: 5JQY)[1] reveals that the conformations of those wt AspH residues directly engaged in NOG/2OG binding (*i.e.* S668, R688, H690, and R735)[1,6] do not alter substantially upon substituting H679 for alanine, even though electron density for a metal ion is not observed. The conformation of H725, whose side chain imidazole engages in metal binding, does not alter substantially upon substituting H679 by alanine; (**c**) superimposition of the hFX-EGFD1$_{86-124}$-4Ser substrate peptide from the H679A AspH:hFX-EGFD1$_{86-124}$-4Ser structure with the one from the reported AspH:Mn:NOG:hFX-EGFD1$_{86-124}$-4Ser structure (cyan: carbon-backbone of the hFX-EGFD1$_{86-124}$-4Ser peptide; PDB ID: 5JQY)[1] reveals similar peptide conformations (Cα RMSD = 0.700 Å), in particular of the amino acids forming the disulfide bridged (Cys101$_{hFX}$ and Cys110$_{hFX}$) ten-membered non-canonical EGFD macrocycle. Note that the Asp103$_{hFX}$ side-chain carboxylate from the reported AspH:Mn:NOG:hFX-EGFD1$_{86-124}$-4Ser structure adopts two conformations.[1]

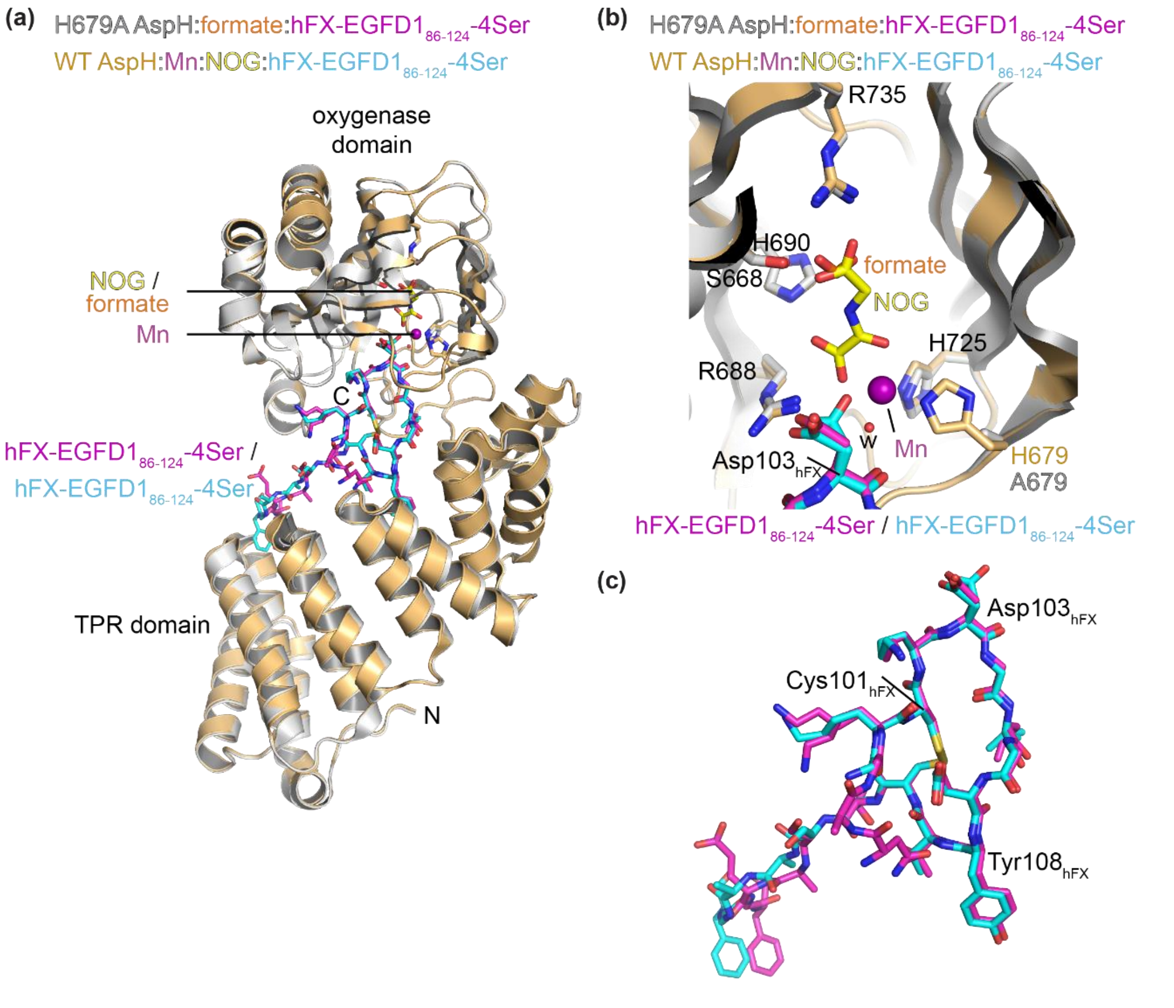

Supporting Figure S15. Views from a crystal structure of H725A AspH complexed with propionate at the active site and a synthetic EGFD substrate peptide (H725A AspH:hFX-EGFD1$_{86\text{-}124}$-4Ser; PDB ID: 7E6J). Colours: grey: H725A His$_6$-AspH$_{315\text{-}758}$; slate blue: carbon-backbone of propionate; magenta: carbon-backbone of the hFX-EGFD1$_{86\text{-}124}$-4Ser peptide (Supporting Figure S2a); red: oxygen; blue: nitrogen.

(**a**) Overview of the H725A AspH:hFX-EGFD1$_{86\text{-}124}$-4Ser crystal structure (1.9 Å resolution). Note, electron density for a metal ion bound to the H725A AspH active site was not observed; (**b**) OMIT electron density map ($mF_o - DF_c$) contoured to 3σ around the hFX-EGFD1$_{86\text{-}124}$-4Ser peptide from the H725A AspH:hFX-EGFD1$_{86\text{-}124}$-4Ser structure reveals electron density for residues Lys100$_{hFX}$ to Thr111$_{hFX}$ including for the disulfide bridged (Cys101$_{hFX}$ and Cys110$_{hFX}$) ten-membered non-canonical EGFD macrocycle. However, electron density is not observed for the side chain of Tyr108$_{hFX}$, an EGFD residue which is part of the AspH-substrate consensus sequence requirement and which has been reported to interact with the TPR domain of wt AspH.[1]

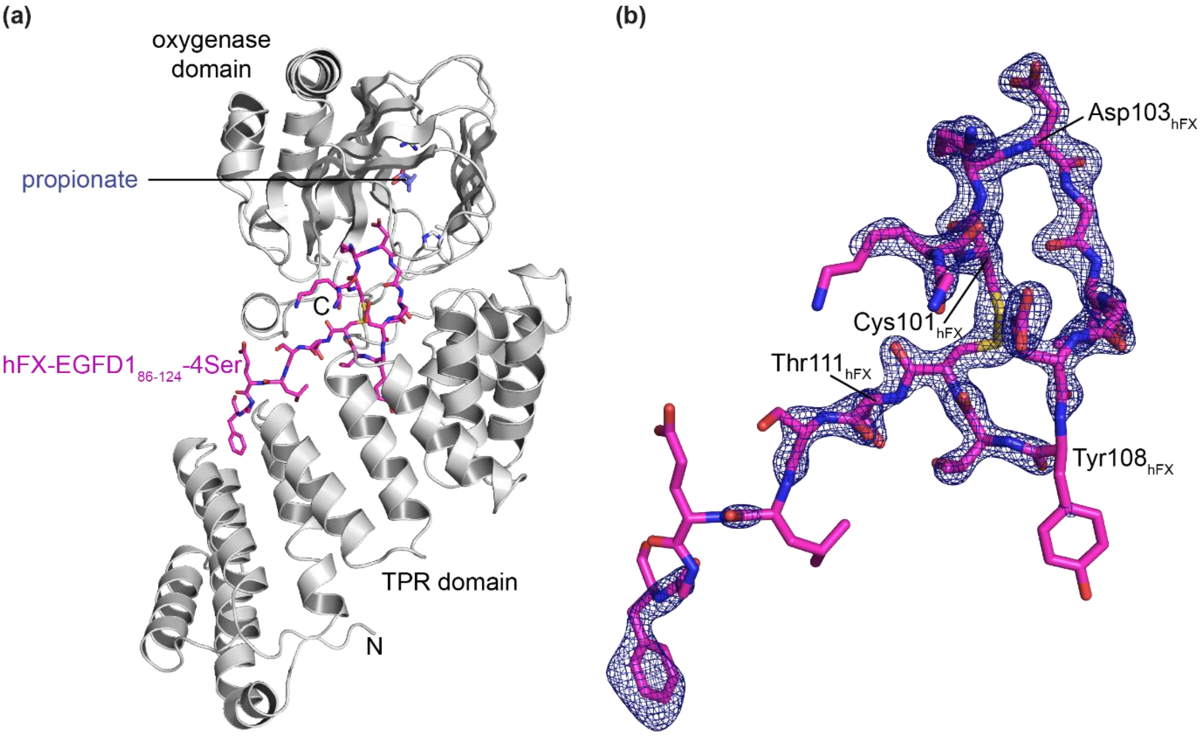

Supporting Figure S16. The substitution of H725 by alanine neither substantially alters the overall AspH fold nor the conformation of key active site residues in the presence of a substrate peptide. Colours: grey: H725A His$_6$-AspH$_{315\text{-}758}$; slate blue: carbon-backbone of propionate; magenta: carbon-backbone of the hFX-EGFD1$_{86\text{-}124}$-4Ser peptide (Supporting Figure S2a); red: oxygen; blue: nitrogen. w: water.

(**a**) Superimposition of a view from the H725A AspH:hFX-EGFD1$_{86\text{-}124}$-4Ser structure (Supporting Figure S15) with one from the reported AspH:Mn:NOG:hFX-EGFD1$_{86\text{-}124}$-4Ser structure (bronze: wt AspH; yellow: carbon-backbone of NOG; cyan: carbon-backbone of the hFX-EGFD1$_{86\text{-}124}$-4Ser peptide; violet: Mn; PDB ID: 5JQY)[1] reveals similar AspH conformations (Cα RMSD = 0.207 Å); (**b**) superimposition of a view from the active site of the H725A AspH:hFX-EGFD1$_{86\text{-}124}$-4Ser structure with one from the reported AspH:Mn:NOG:hFX-EGFD1$_{86\text{-}124}$-4Ser structure (bronze: wt AspH; yellow: carbon-backbone of NOG; cyan: carbon-backbone of the hFX-EGFD1$_{86\text{-}124}$-4Ser peptide; violet: Mn; PDB ID: 5JQY)[1] reveals that the conformations of those wt AspH residues directly engaged in NOG/2OG binding (*i.e.* S668, R688, H690, and R735)[1,6] do not alter substantially upon substituting H725 by alanine, even though electron density for a metal ion is not observed. The conformation of H679, whose side chain imidazole engages in metal binding, does not alter substantially upon substituting H725 for alanine; (**c**) superimposition of the hFX-EGFD1$_{86\text{-}124}$-4Ser substrate peptide from the H725A AspH:hFX-EGFD1$_{86\text{-}124}$-4Ser structure with the one from the reported AspH:Mn:NOG:hFX-EGFD1$_{86\text{-}124}$-4Ser structure (cyan: carbon-backbone of the hFX-EGFD1$_{86\text{-}124}$-4Ser peptide; PDB ID: 5JQY)[1] reveals similar peptide conformations (Cα RMSD = 0.266 Å), in particular of the amino acids forming the disulfide bridged (Cys101$_{hFX}$ and Cys110$_{hFX}$) ten-membered non-canonical EGFD macrocycle. Note that the Asp103$_{hFX}$ side-chain carboxylate from the reported AspH:Mn:NOG:hFX-EGFD1$_{86\text{-}124}$-4Ser structure adopts two conformations.[1]

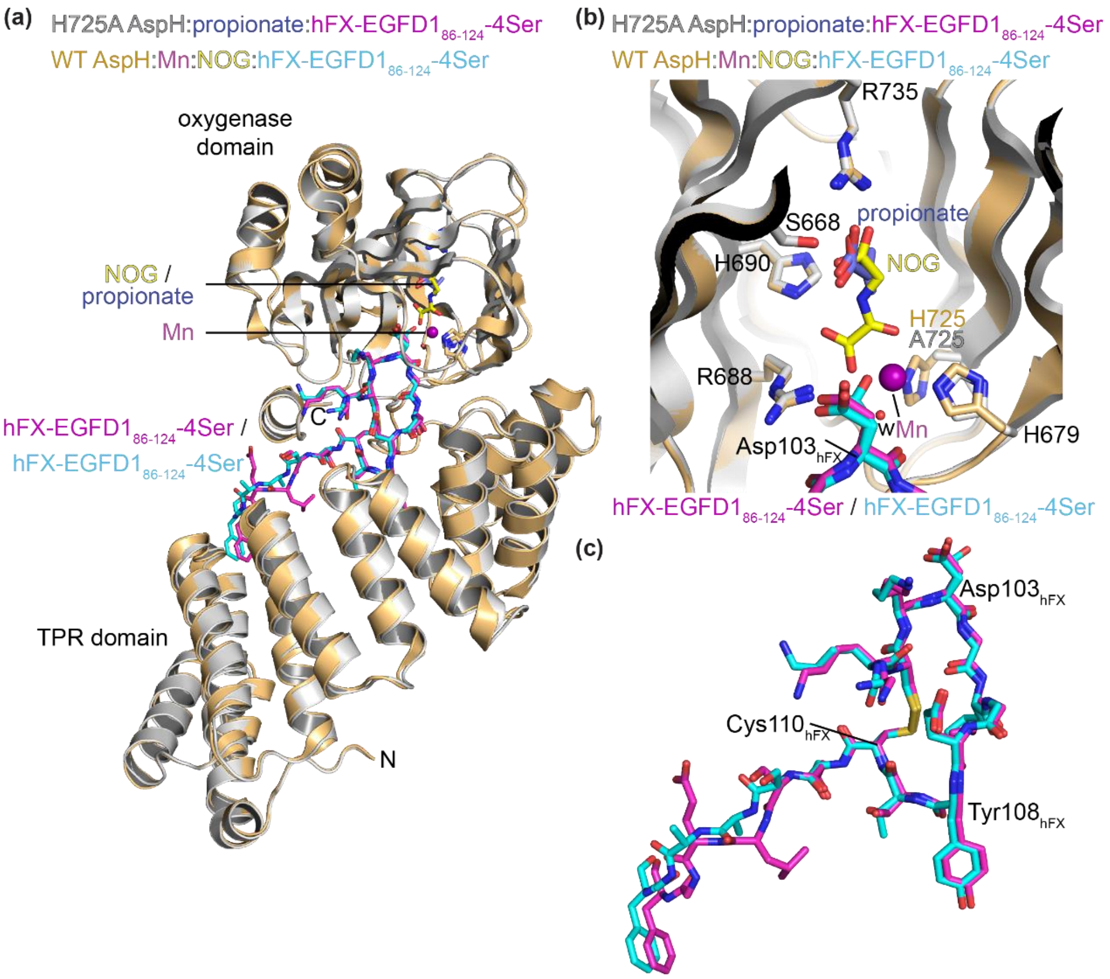

Supporting Figure S17. The overall folds and conformations of the H725 and H679A AspH variants are similar. Colours: grey: H725A His$_6$-AspH$_{315-758}$; slate blue: carbon-backbone of propionate; magenta: carbon-backbone of the hFX-EGFD1$_{86-124}$-4Ser peptide (Supporting Figure S2a); red: oxygen; blue: nitrogen.

(**a**) Superimposition of a view from the H725A AspH:hFX-EGFD1$_{86-124}$-4Ser structure (Supporting Figure S15) with one from the H679A AspH:hFX-EGFD1$_{86-124}$-4Ser structure (pale yellow: H679A His$_6$-AspH$_{315-758}$; orange: carbon-backbone of formate; dark green: carbon-backbone of the hFX-EGFD1$_{86-124}$-4Ser peptide; Supporting Figure S12) reveals similar AspH conformations (Cα RMSD = 0.268 Å); (**b**) superimposition of a view from the active site of the H725A AspH:hFX-EGFD1$_{86-124}$-4Ser structure with one from the H679A AspH:hFX-EGFD1$_{86-124}$-4Ser structure (pale yellow: H679A His$_6$-AspH$_{315-758}$; orange: carbon-backbone of formate; dark green: carbon-backbone of the hFX-EGFD1$_{86-124}$-4Ser peptide; Supporting Figure S12) reveals that the conformations of those wt AspH residues directly engaged in NOG/2OG binding (*i.e.* S668, R688, H690, and R735)[1,6] do not alter substantially if either H679 or H725 were substituted for alanine, even though electron density for a metal ion is not observed; (**c**) superimposition of the hFX-EGFD1$_{86-124}$-4Ser substrate peptide from the H725A AspH:hFX-EGFD1$_{86-124}$-4Ser structure with the one from the H679A AspH:hFX-EGFD1$_{86-124}$-4Ser structure (dark green: carbon-backbone of the hFX-EGFD1$_{86-124}$-4Ser peptide; Supporting Figure S12) reveals similar peptide conformations (Cα RMSD = 0.076 Å), in particular of the amino acids forming the disulfide bridged (Cys101$_{hFX}$ and Cys110$_{hFX}$) ten-membered non-canonical EGFD macrocycle.

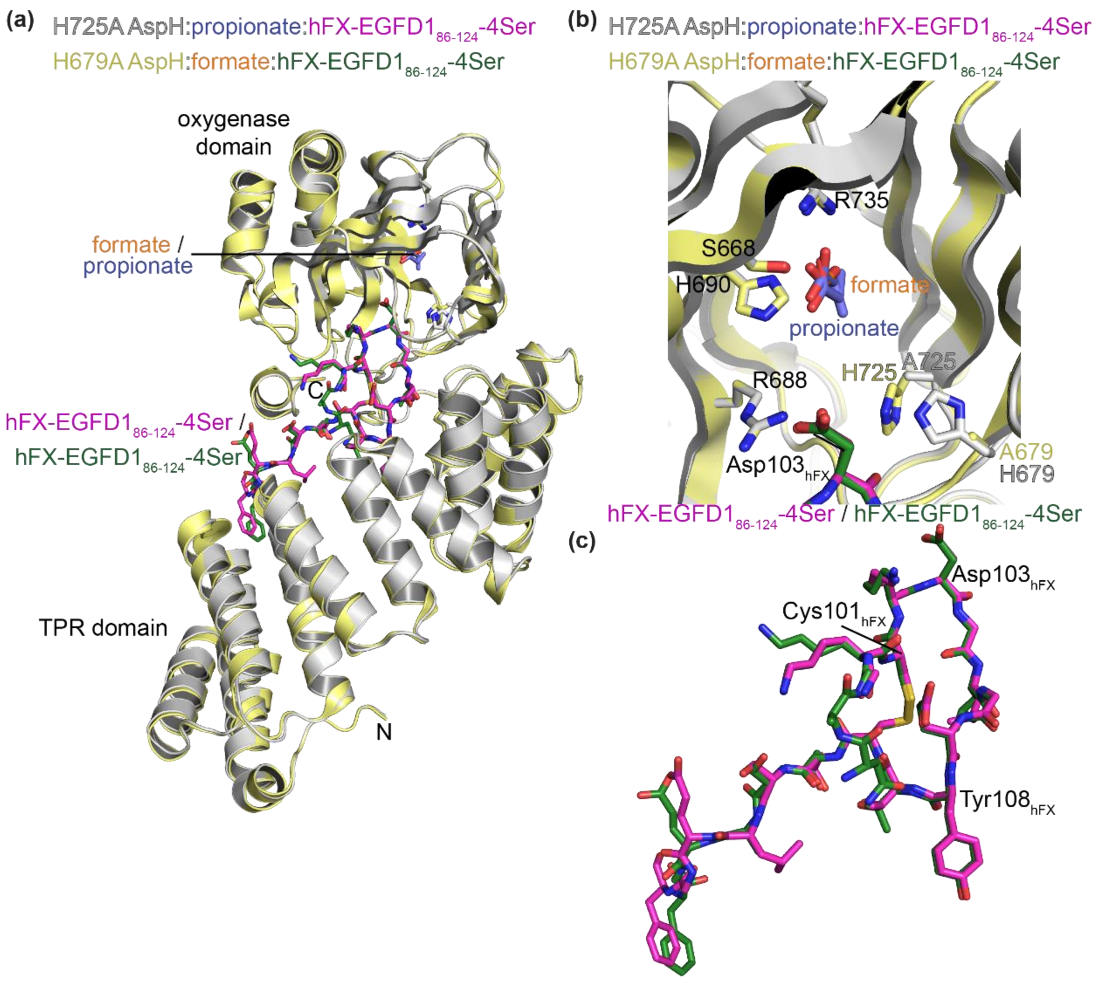

Supporting Figure S18. Views from a crystal structure of H679A AspH complexed with Mn, NOG, and a synthetic EGFD substrate peptide (H679A AspH:Mn:NOG:hFX-EGFD1$_{86-124}$-4Ser; PDB ID: 6Q9F). Colours: grey: H679A His$_6$-AspH$_{315-758}$; yellow: carbon-backbone of *N*-oxalylglycine (NOG); violet: Mn; magenta: carbon-backbone of the hFX-EGFD1$_{86-124}$-4Ser peptide (Supporting Figure S2a); red: oxygen; blue: nitrogen.

(**a**) Overview of the H679A AspH:NOG:hFX-EGFD1$_{86-124}$-4Ser crystal structure (1.6 Å resolution); (**b**) OMIT electron density map (mF$_o$−DF$_c$) contoured to 3σ around the hFX-EGFD1$_{86-124}$-4Ser peptide from the H679A AspH:Mn:NOG:hFX-EGFD1$_{86-124}$-4Ser structure reveals electron density for residues Lys100$_{hFX}$ to Thr111$_{hFX}$ including for the disulfide bridged (Cys101$_{hFX}$ and Cys110$_{hFX}$) ten-membered non-canonical EGFD macrocycle. Incomplete electron density is observed for the side chain of Tyr108$_{hFX}$, an EGFD residue which is part of the AspH-substrate consensus sequence requirement. In reported AspH:substrate complex structures, the side chain of Tyr108$_{hFX}$ has been shown to interact with the TPR domain of AspH.[1]

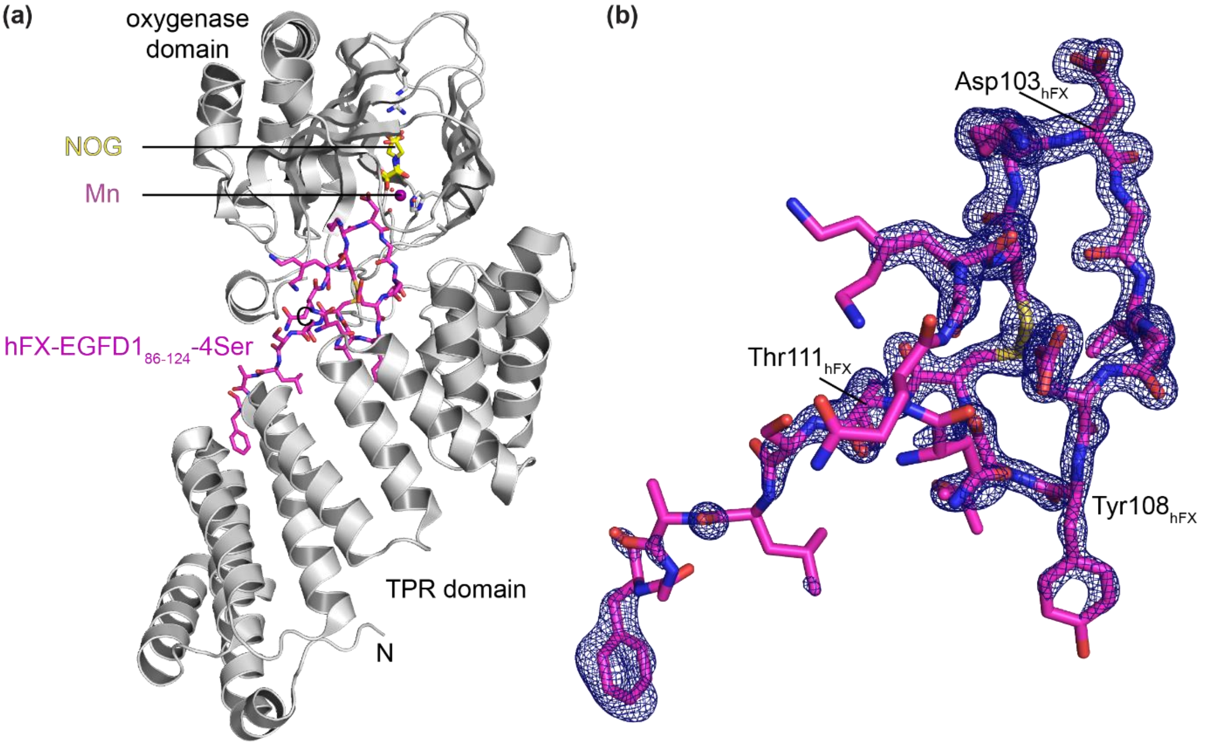

Supporting Figure S19. The substitution of formate in the active site of the H679A AspH:hFX-EGFD1$_{86-124}$-4Ser structure for NOG and Mn neither substantially alters the overall AspH fold nor the conformation of key active site residues. Colours: grey: H679A His$_6$-AspH$_{315-758}$; yellow: carbon-backbone of *N*-oxalylglycine (NOG); violet: Mn; magenta: carbon-backbone of the hFX-EGFD1$_{86-124}$-4Ser peptide (Supporting Figure S2a); red: oxygen; blue: nitrogen. w: water.

(**a**) Superimposition of a view from the H679A AspH:Mn:NOG:hFX-EGFD1$_{86-124}$-4Ser structure (Supporting Figure S18) with one from the H679A AspH:hFX-EGFD1$_{86-124}$-4Ser structure (olive: H679A AspH; orange: carbon-backbone of formate; marine blue: carbon-backbone of the hFX-EGFD1$_{86-124}$-4Ser peptide; Supporting Figure S12) reveals similar AspH conformations (Cα RMSD = 0.253 Å); (**b**) superimposition of a view from the active site of the H679A AspH:Mn:NOG:hFX-EGFD1$_{86-124}$-4Ser structure with one from the H679A AspH:hFX-EGFD1$_{86-124}$-4Ser structure (olive: H679A AspH; orange: carbon-backbone of formate; marine blue: carbon-backbone of the hFX-EGFD1$_{86-124}$-4Ser peptide; Supporting Figure S12) reveals that the conformations of those wt AspH residues directly engaged in NOG/2OG binding (*i.e.* S668, R688, H690, and R735)[1,6] do not alter substantially upon substituting formate by NOG and Mn (note that the NOG glycine unit adopts two conformations, one of which superimposes with the formate in the H679A AspH:hFX-EGFD1$_{86-124}$-4Ser structure). The conformation of H725 does not alter substantially upon coordination of a Mn ion by the side chain imidazole. (**c**) superimposition of the hFX-EGFD1$_{86-124}$-4Ser substrate peptide from the H679A AspH:Mn:NOG:hFX-EGFD1$_{86-124}$-4Ser structure with the one from the H679A AspH:hFX-EGFD1$_{86-124}$-4Ser structure (marine blue: carbon-backbone of the hFX-EGFD1$_{86-124}$-4Ser peptide) reveals similar peptide conformations (Cα RMSD = 0.17 Å), in particular of the amino acids forming the disulfide bridged (Cys101$_{hFX}$ and Cys110$_{hFX}$) ten-membered non-canonical EGFD macrocycle.

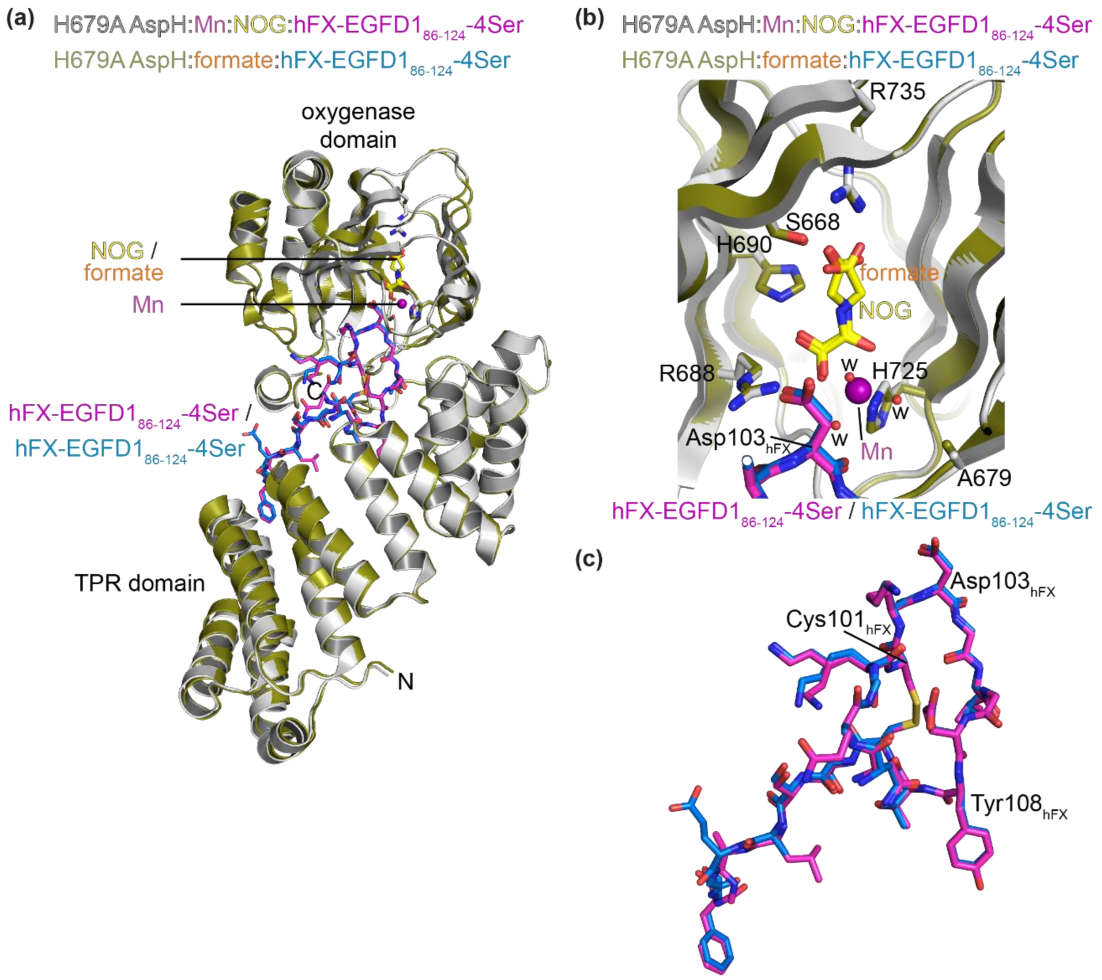

Supporting Figure S20. The substitution of H679 by alanine neither substantially alters the overall AspH fold nor the conformation of key active site residues in the presence of a substrate peptide. Colours: grey: H679A His$_6$-AspH$_{315-758}$; yellow: carbon-backbone of *N*-oxalylglycine (NOG); violet: Mn; magenta: carbon-backbone of the hFX-EGFD1$_{86-124}$-4Ser peptide (Supporting Figure S2a); red: oxygen; blue: nitrogen. w: water. (**a**) Superimposition of a view from the H679A AspH:Mn:NOG:hFX-EGFD1$_{86-124}$-4Ser structure (Supporting Figure S18) with one from the reported wt AspH:Mn:NOG:hFX-EGFD1$_{86-124}$-4Ser structure (bronze: wt AspH; maroon: carbon-backbone of NOG; cyan: carbon-backbone of the hFX-EGFD1$_{86-124}$-4Ser peptide; lavender blue: Mn; PDB ID: 5JQY)[1] reveals similar AspH conformations (Cα RMSD = 0.235 Å); (**b**) superimposition of the hFX-EGFD1$_{86-124}$-4Ser substrate peptide from the H679A AspH:Mn:NOG:hFX-EGFD1$_{86-124}$-4Ser structure with the one from the reported wt AspH:Mn:NOG:hFX-EGFD1$_{86-124}$-4Ser structure (cyan: carbon-backbone of the hFX-EGFD1$_{86-124}$-4Ser peptide; PDB ID: 5JQY)[1] reveals similar peptide conformations (Cα RMSD = 0.754 Å), in particular of the amino acids forming the disulfide bridged (Cys101$_{hFX}$ and Cys110$_{hFX}$) ten-membered non-canonical EGFD macrocycle. Note that the Asp103$_{hFX}$ side-chain carboxylate from the reported AspH:Mn:NOG:hFX-EGFD1$_{86-124}$-4Ser structure adopts two conformations.[1]

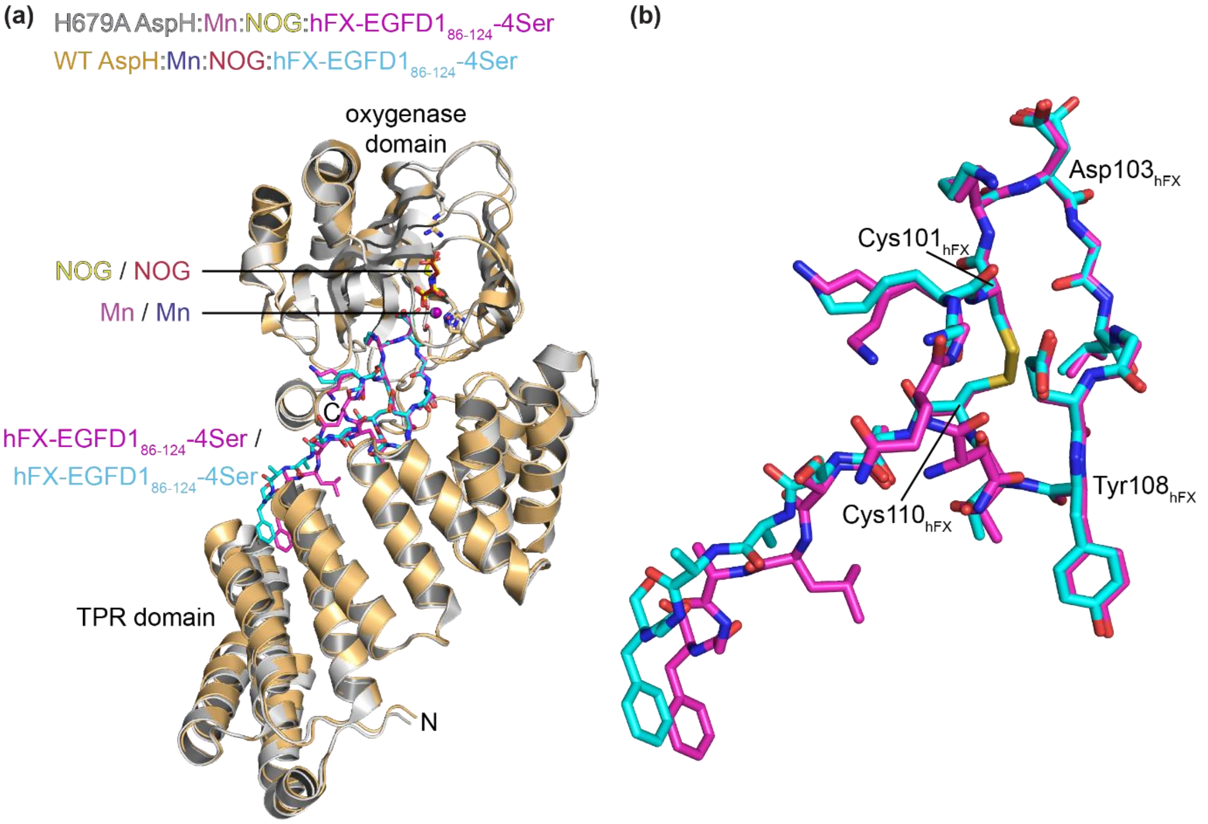

Supporting Figure S21. Proposed outline catalytic cycle for the AspH-catalysed hydroxylation of EGFD Asp- and Asn-residues. Based on reported wt AspH:2OG and wt AspH:2OG:substrate structures[6] and mechanistic studies with other 2OG oxygenases,[7] an outline catalytic cycle for AspH has been proposed.[6] Initially, 2OG coordinates to the AspH Fe(II) cofactor in a bidentate manner through one of its C1-carboxylate oxygen atoms and its C2-ketone oxygen atom (**B**), replacing two ligating water molecules in the Fe(II) complex **A**. The 2OG C1-carboxylate oxygen atom coordinates Fe(II) *trans* to His679 (red).[6] The EGFD substrate then binds AspH with the Asp/Asn-residue undergoing β-hydroxylation displacing the water molecule (blue) binding Fe(II) *trans* to His725 (blue, **C**);[6] however, in the H679A AspH:NOG:hFX-EGFD1$_{86-124}$-4Ser structure, three water molecules are still coordinated to the metal ion indicating the substrate binding occurs prior to water release. Interactions of the Asp/Asn side chain carboxylate/amide with the active site Fe(II) are likely weak, as several different conformations for the Asp/Asn side chains are observed in the crystalline state.[1,6] Molecular O_2 likely binds to Fe(II) *trans* to the His725 (blue) replacing the substrate side chain carboxylate to give the superoxo-Fe(III) complex **D**. Irreversible oxidative decarboxylation of 2OG occurs, potentially via a peroxyester intermediate,[7b] liberating CO_2 and forming an Fe(IV)-oxo:succinate:substrate complex (*i.e.* the ferryl intermediate, **E**). The *pro-R* hydrogen of the substrate EGFD Asn/Asp-side chain is proximate to the Fe(IV)-oxo moiety of complex **E** and is hydroxylated,[1,6] via direct insertion or a radical mechanism proceeding via an Fe(III) intermediate, to afford the Fe(II):succinate:hydroxylated substrate complex **F**. Finally, three water molecules replace the oxidized EGFD substrate and succinate to reform complex **A**. Note, that the role of the water-occupied coordination site *trans* to the 2OG ketone oxygen is not addressed in this mechanistic proposal; it is likely, this is important as it distinguishes AspH from other 2OG oxygenases;[7] thus, other mechanistic scenarios are feasible.

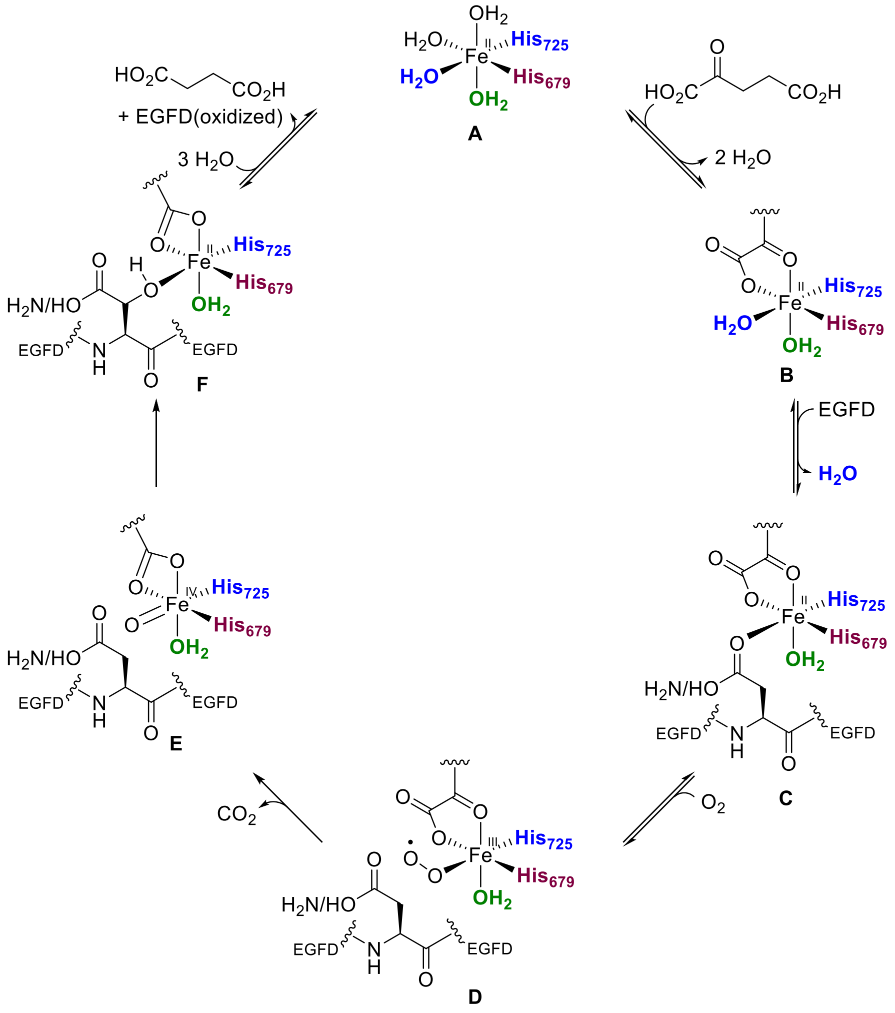

Supporting Figure S22. Human Aspartate β-hydroxylase domain-containing protein 1 is predicted to bear an unusual Fe(II) binding site composed of an Arg- and a His-residue. (**a**) Sequence alignment of human wt AspH (top) with human wt aspartate β-hydroxylase domain-containing protein 1 (AspHD1, bottom) suggests that AspHD1 bears an unusual Fe(II) binding site composed of an Arg- and a His-residue, *i.e.* R304 and H349. Sequences were obtained from the National Center for Biotechnology Information (NCBI) database[8] and processed using GeneDoc 2.7;[9] black shading indicates high sequence conservation; (predicted) metal binding residues are in red (*i.e.* H679 and H725 for wt AspH and R304 and H349 for wt AspHD1); Gly-residues substituting the typical Glu- or Asp-residues present in the Fe(II)-binding facial triad of 2OG hydroxylases are in blue; (**b** and **c**) superimposition of a view from the wt AspH:2OG structure (grey: wt AspH; green: carbon-backbone of 2OG; violet: Mn; PDB ID: 6YYU[6]) with one from the predicted structure of AspHD1 (red: wt AspHD1) which was calculated using the transform-restrained Rosetta software package.[10] Close-up (**c**) of a view from the predicted AspHD1 metal binding site reveals how the side chains of R304 and H349 may interact with the active site metal. Note that in the absence of metal, the side chain of R304 is predicted to orient away from the AspHD1 active site.

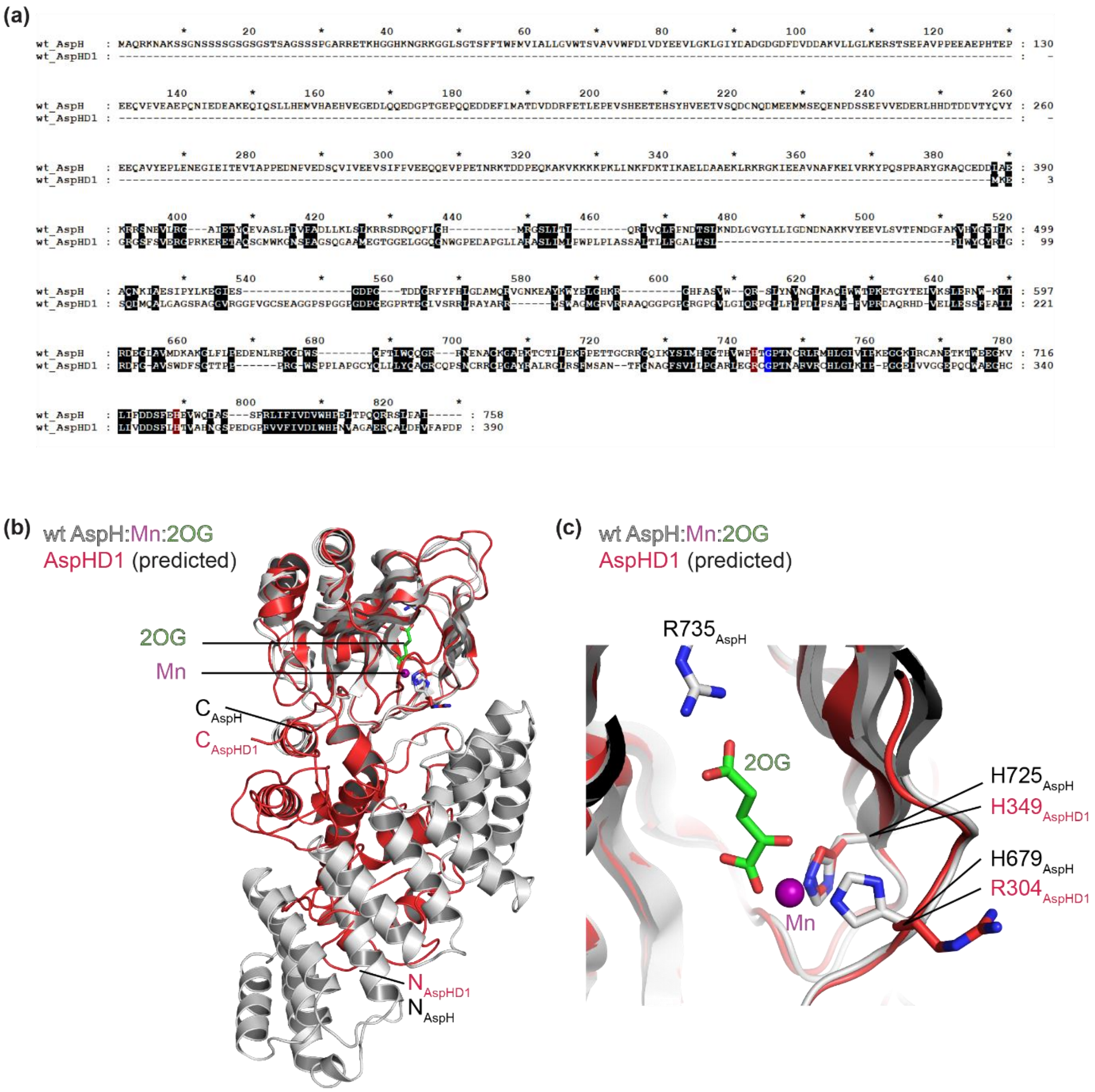

Supporting Table S1. Crystallization conditions, data collection, and refinement statistics for the H679A and H725A AspH variant complexes.[a)]

	H725A His$_6$-AspH$_{315-758}$·hFX-EGFD1$_{86-124}$-4Ser (H725A AspH:hFX-EGFD1$_{86-124}$-4Ser)	H679A His$_6$-AspH$_{315-758}$·Mn^{II}·NOG·hFX-EGFD1$_{86-124}$-4Ser (H679A AspH:Mn:NOG:hFX-EGFD1$_{86-124}$-4Ser)	H679A His$_6$-AspH$_{315-758}$·hFX-EGFD1$_{86-124}$-4Ser (H679A AspH:hFX-EGFD1$_{86-124}$-4Ser)	H679A His$_6$-AspH$_{315-758}$ (H679A AspH:acetate)
**PDB ID**	7E6J	6Q9F	6Q9I	6QA5
**Crystallization**				
Precipitation conditions	18 mg/mL His$_6$-H725A-AspH$_{315-758}$ (330 μM), 100 mM PCTP, pH 7.0, 25% w/v PEG1500, 726 μM hFX-EGFD1$_{86-124}$-4Ser	18 mg/mL His$_6$-H679A-AspH$_{315-758}$ (330 μM), 1 mM MnCl$_2$, 2 mM NOG, 0.1 M Tris-HCl, pH 8.0, 0.2 M sodium chloride, 20% w/v PEG6000, 726 μM hFX-EGFD1$_{86-124}$-4Ser	18 mg/mL His$_6$-H679A-AspH$_{315-758}$ (330 μM), 0.2 M sodium formate, pH 7.5, 20% w/v PEG3350, 726 μM hFX-EGFD1$_{86-124}$-4Ser	18 mg/mL His$_6$-H679A-AspH$_{315-758}$ (330 μM), 0.1 M PCTP, pH 9.0, 0.2 M sodium acetate, 25% w/v PEG1500
**Data collection**				
Space group	$P2_12_12_1$	$P2_12_12_1$	$P2_12_12_1$	$P2_12_12_1$
Symmetry	orthorhombic	orthorhombic	orthorhombic	orthorhombic
Cell dimensions:				
a, *b*, *c* (Å)	50.24, 86.84, 123.54	49.81, 91.54, 122.90	50.13, 91.36, 123.54	48.78, 70.52, 173.50
α, β, γ (°)	90.00, 90.00, 90.00	90.00, 90.00, 90.00	90.00, 90.00, 90.00	90.00, 90.00, 90.00
X-Ray source[b)]	Synchrotron (DLS I03)	Synchrotron (DLS I04)	Synchrotron (DLS I04)	Synchrotron (DLS I04)
Resolution (Å)[c)]	46.54-1.90 (1.93-1.90)	46.16-1.63 (1.69-1.63)	46.45-1.85 (1.92-1.85)	44.72-2.65 (2.74-2.65)
R_{merge}	0.153 (1.792)	0.061 (1.000)	0.077 (1.000)	0.241 (1.319)
I / σI	10.5 (0.8)	35.6 (1.9)	27.1 (1.6)	9.0 (2.0)
CC (1/2)	0.998 (0.699)	0.999 (0.891)	0.998 (0.705)	0.997 (0.608)
Total number unique reflections	43495 (2159)	70950 (3465)	49046 (2451)	18051 (884)
Completeness (%)	100.0 (99.3)	100.0 (100.0)	100.0 (100.0)	100.0 (100.0)
Multiplicity	13.3 (13.7)	12.9 (13.0)	9.8 (9.5)	12.9 (13.3)
**Refinement**				
R_{work} / R_{free}	0.170 / 0.213	0.182 / 0.200	0.202 / 0.223	0.197 / 0.224
No. atoms:	3992	4010	3923	3514
B-factors (Å^2):	43.0	46.0	59.0	50.0
R.m.s. deviations:				
Bond lengths (Å)	0.002	0.012	0.007	0.005
Bond angles (°)	0.571	1.199	0.973	0.775

[a)]Experimental details are specified in the Supplementary Information (Section 3); [b)]DLS: Diamond Light Source; [c)]Values in parentheses are for highest-resolution shell.

2. AspH variant inhibition assays

Solutions of the inhibitors (100% DMSO) were dry dispensed across 384-well polypropylene assay plates (Greiner) in an approximately three-fold and 11-point dilution series (100 μM top concentration) using an ECHO 550 acoustic dispenser (Labcyte). DMSO and 2,4-PDCA were used as negative and positive inhibition controls, respectively. The final DMSO concentration was kept constant at 0.5%$_{v/v}$ throughout all experiments (using the DMSO backfill option of the acoustic dispenser). Each reaction was performed in technical duplicates in adjacent wells of the assay plates; additionally, assays were performed in two independent duplicates. Cosubstrate/cofactor stock solutions (L-ascorbic acid, LAA: 50 mM in Milli-Q® Ultrapure water; 2-oxoglutarate, 2OG: 10 mM in Milli-Q® Ultrapure water; ammonium iron(II) sulfate hexahydrate, FAS, $(NH_4)_2Fe(SO_4)_2 \cdot 6H_2O$: 400 mM in 20 mM HCl diluted to 1 mM in Milli-Q® Ultrapure water) were freshly prepared each day from commercially sourced solids (Sigma Aldrich).

The Enzyme Mixture (25 μL/well), containing H679A or H725A His$_6$-AspH$_{315-758}$ variant (0.2 μM) in 25 mM HEPES buffer (pH 7.5), was dispensed across the inhibitor-containing 384-well assay plates with a multidrop dispenser (ThermoFischer Scientific) at 20°C under an ambient atmosphere. The plates were subsequently centrifuged (1000 rpm, 10 s) and incubated for 15 minutes at 20°C. The Substrate Mixture (25 μL/well), containing hFX-EGFD1$_{86-124}$-4Ser peptide (8.0 μM; Supporting Figure 2a), LAA (200 μM), 2OG (for H679A: 220 μM; for H725A: 430 μM), and FAS (for H679A: 8.0 μM; for H725A: 20 μM) in 25 mM HEPES buffer (pH 7.5), was added using the multidrop dispenser. The plates were centrifuged (1000 rpm, 10 s) and after incubating (22 min for H679A; 37 min for H725A), the enzyme reaction was stopped by addition of 10%$_{v/v}$ aqueous formic acid (5 μL/well). The plates were then centrifuged (1000 rpm, 20 s) and analysed by MS.

MS-analyses were performed using a RapidFire RF 365 high-throughput sampling robot (Agilent) attached to an iFunnel Agilent 6550 accurate mass quadrupole time-of-flight (Q-TOF) mass spectrometer operated in the positive ionization mode. Assay samples were aspirated under vacuum for 0.6 s and loaded onto a C4 solid phase extraction (SPE) cartridge. After loading, the C4 SPE cartridge was washed with 0.1%$_{v/v}$ aqueous formic acid to remove non-volatile buffer salts (5.5 s, 1.5 mL/min). The peptide was eluted from the SPE cartridge with 0.1%$_{v/v}$ aqueous formic acid in 80/20$_{v/v}$ acetonitrile/water into the mass spectrometer (5.5 s, 1.5 mL/min) and the SPE cartridge re-equilibrated with 0.1%$_{v/v}$ aqueous formic acid (0.5 s, 1.25 mL/min). The mass spectrometer parameters were: capillary voltage (4000 V), nozzle voltage (1000 V), fragmentor voltage (365 V), gas temperature (280°C), gas flow (13 L/min), sheath gas temperature (350°C), sheath gas flow (12 L/min). For data analysis, the m/z +4 charge states of the hFX-EGFD1$_{86-124}$-4Ser peptide (substrate) and the hydroxylated hFX-EGFD1$_{86-124}$-4Ser peptide (product) were used to extract ion chromatogram data; peak areas were integrated using RapidFire Integrator software (Agilent). Data were exported into Microsoft Excel and used to calculate the % conversion of the hydroxylation reaction using the equation: % conversion = 100 x (integral hydroxylated hFX-EGFD1$_{86-124}$-4Ser peptide) / (integral substrate hFX-EGFD1$_{86-124}$-4Ser peptide + integral hydroxylated hFX-EGFD1$_{86-124}$-4Ser peptide). Normalized dose-response curves (2,4-PDCA and DMSO controls) were obtained from the raw data by non-linear regression (GraphPad Prism 5) and used to determine IC_{50}-values. The standard deviation (SD) of two independent IC_{50} determinations (n = 2) was calculated using GraphPad Prism 5. Z'-factors were calculated according to the literature using Microsoft Excel.[5]

3. Crystallography

High-throughput crystallization experiments were performed in 96-well, 3-subwell, low profile Intelliplates (Art Robbins Instruments) using a Phoenix RE liquid dispensing robot (Art Robbins Instruments) and Hampton Research (PEG/Ion, Crystal Screen) or Molecular Dimensions (PACT Premier) crystallization screens. *N*-Terminally His_6-tagged $AspH_{315-758}$ variants (18 mg/mL in 50 mM HEPES buffer, pH 7.5) were mixed with 1 mM $MnCl_2$, 2 mM 2OG or NOG, and, where appropriate, the hFX-EGFD1_{86-124}-4Ser peptide as an AspH substrate (note that $MnCl_2$ and 2OG/NOG were present all crystallization experiments, including those for which no metal/ligand-bound structure was obtained). Crystals were grown using the vapour diffusion method at 4°C in 200 nL or 300 nL sitting drops with 2:1, 1:1 or 1:2 sample:well solution ratios; precipitants are listed in the Supporting Table S1. Crystals were cryo-protected using mother liquor supplemented with $25\%_{v/v}$ glycerol before cryo-cooling in liquid N_2. Data were collected at 100 K using synchrotron radiation at Diamond Light Source (DLS) beamlines I03 and I04. For the H679A AspH structures, data were indexed, integrated, and scaled using the HKL2000 program suite;[11] for the H725A AspH:hFX-EGFD1_{86-124}-4Ser structure, data were indexed, integrated, and scaled using the Xia2[12] strategy of the beamline auto-processing pipeline (Supporting Table S1). The AspH crystal structures were determined by molecular replacement (MR) using the AutoMR (PHASER[13]) subroutine in PHENIX[14]. The search model used for MR was based on PDB ID 5JTC[15] for the H725A AspH:hFX-EGFD1_{86-124}-4Ser structure, PDB ID 5JQY[1] for the H679A AspH:hFX-EGFD1_{86-124}-4Ser structure, and PDB ID 6Q9F for all other H679A AspH structures. The structural model was improved by iterative cycles of manual re-building in COOT[16] and crystallographic refinement in phenix.refine[17] (refinement details are summarized in Supporting Table S1).

Crystal structure data for the *N*-terminal His_6-tagged $AspH_{315-758}$ variants H679A and H725A complexed to carboxylic acids or NOG, and, in some cases, substrate peptide (hFX-EGFD1_{86-124}-4Ser) and Mn, are deposited in the protein data bank with PDB accession codes: 7E6J (H725A AspH:hFX-EGFD1_{86-124}-4Ser), 6Q9F (H679A AspH:Mn:NOG:hFX-$EGFD_{86-124}$-4Ser), 6Q9I (H679A AspH:hFX-$EGFD_{86-124}$-4Ser), and 6QA5 (H679A AspH:acetate). PyMOL[18] was used for the generation of graphical representations; polder omit maps were calculated using Polder Maps[19] in PHENIX[14].

4. References

[1] I. Pfeffer, L. Brewitz, T. Krojer, S. A. Jensen, G. T. Kochan, N. J. Kershaw, K. S. Hewitson, L. A. McNeill, H. Kramer, M. Münzel, R. J. Hopkinson, U. Oppermann, P. A. Handford, M. A. McDonough, C. J. Schofield, *Nat. Commun.* **2019**, *10*, 4910.

[2] a) K. F. Geoghegan, H. B. F. Dixon, P. J. Rosner, L. R. Hoth, A. J. Lanzetti, K. A. Borzilleri, E. S. Marr, L. H. Pezzullo, L. B. Martin, P. K. LeMotte, A. S. McColl, A. V. Kamath, J. G. Stroh, *Anal. Chem.* **1999**, *267*, 169-184; b) Z. Yan, G. W. Caldwell, P. A. McDonell, *Biochem. Biophys. Res. Commun.* **1999**, *262*, 793-800.

[3] a) P. Fernlund, J. Stenflo, *J. Biol. Chem.* **1983**, *258*, 12509-12512; b) B. A. McMullen, K. Fujikawa, W. Kisiel, T. Sasagawa, W. N. Howald, E. Y. Kwa, B. Weinstein, *Biochemistry* **1983**, *22*, 2875-2884.

[4] L. Brewitz, A. Tumber, C. J. Schofield, *J. Biol. Chem.* **2020**, *295*, 7826-7838.

[5] J.-H. Zhang, T. D. Y. Chung, K. R. Oldenburg, *J. Biomol. Screen.* **1999**, *4*, 67-73.

[6] L. Brewitz, Y. Nakashima, C. J. Schofield, *Chem. Sci.* **2021**, *12*, 1327-1342.

[7] a) H. M. Hanauske-Abel, V. Günzler, *J. Theor. Biol.* **1982**, *94*, 421-455; b) S. Martinez, R. P. Hausinger, *J. Biol. Chem.* **2015**, *290*, 20702-20711.

[8] L. Y. Geer, A. Marchler-Bauer, R. C. Geer, L. Han, J. He, S. He, C. Liu, W. Shi, S. H. Bryant, *Nucleic Acids Res.* **2010**, *38*, D492-D496.

[9] K. B. Nicholas, H. B. Nicholas Jr., D. W. Deerfield II., *embnet.news* **1997**, *4*, 1-4.

[10] J. Yang, I. Anishchenko, H. Park, Z. Peng, S. Ovchinnikov, D. Baker, *Proc. Natl. Acad. Sci. USA* **2020**, *117*, 1496-1503.

[11] Z. Otwinowski, W. Minor, in *Methods Enzymol., Vol. 276*, Academic Press, **1997**, pp. 307-326.

[12] G. Winter, *J. Appl. Cryst.* **2010**, *43*, 186-190.

[13] A. J. McCoy, R. W. Grosse-Kunstleve, P. D. Adams, M. D. Winn, L. C. Storoni, R. J. Read, *J. Appl. Cryst.* **2007**, *40*, 658-674.

[14] P. D. Adams, P. V. Afonine, G. Bunkóczi, V. B. Chen, I. W. Davis, N. Echols, J. J. Headd, L.-W. Hung, G. J. Kapral, R. W. Grosse-Kunstleve, A. J. McCoy, N. W. Moriarty, R. Oeffner, R. J. Read, D. C. Richardson, J. S. Richardson, T. C. Terwilliger, P. H. Zwart, *Acta Cryst. D* **2010**, *66*, 213-221.

[15] L. Brewitz, A. Tumber, I. Pfeffer, M. A. McDonough, C. J. Schofield, *Sci. Rep.* **2020**, *10*, 8650.

[16] P. Emsley, B. Lohkamp, W. G. Scott, K. Cowtan, *Acta Cryst. D* **2010**, *66*, 486-501.

[17] P. V. Afonine, R. W. Grosse-Kunstleve, N. Echols, J. J. Headd, N. W. Moriarty, M. Mustyakimov, T. C. Terwilliger, A. Urzhumtsev, P. H. Zwart, P. D. Adams, *Acta Cryst. D* **2012**, *68*, 352-367.

[18] W. L. DeLano, **2002**, De Lano Scientific, San Carlos.

[19] D. Liebschner, P. V. Afonine, N. W. Moriarty, B. K. Poon, O. V. Sobolev, T. C. Terwilliger, P. D. Adams, *Acta Cryst. D* **2017**, *73*, 148-157.
